# Linking microbes to *in situ* methane oxidation rates in a eutrophic freshwater lake

**DOI:** 10.3389/fmicb.2026.1789101

**Published:** 2026-03-06

**Authors:** Jennifer A. Baily, Zachary W. Hudspeth, Joshua L. Morningstar, Howard P. Mendlovitz, Christopher S. Martens, Karen G. Lloyd

**Affiliations:** 1 Department of Microbiology, University of Tennessee, Knoxville, TN, United States; 2 Department of Earth Sciences, University of Southern California, Los Angeles, CA, United States; 3 Department of Earth, Marine and Environmental Sciences, University of North Carolina at Chapel Hill, Chapel Hill, NC, United States

**Keywords:** 16S rRNA gene amplicon analysis, aerobic methane oxidation, aerobic methanotroph, *in situ* incubation, methane, methylotroph

## Abstract

**Introduction:**

Aerobic methanotrophs and non-methanotrophic methylotrophs drive methane cycling in oxic freshwater lakes. Most knowledge about biological aerobic methane oxidation (MOx) comes from *ex situ* rate experiments, laboratory cultures, and static measurements of natural abundances.

**Methods:**

We investigated the link between MOx rate constants measured with a novel *in situ* incubation device and the microbial community in Jordan Lake, a methane-rich freshwater lake in NC, USA. We coupled relative abundances of 16S rRNA genes and quantitative PCR of particulate methane monooxygenase subunit A (*pmoA*) to methane, oxygen, temperature, and *in situ* MOx rate constants, all collected using the novel iBag *in situ* incubation system.

**Results:**

In 16 incubations spread across 13 months, *Methylococcaceae*, whose cultured members are obligate aerobic methanotrophs, strongly and inversely correlate with naturally-varying oxygen but not with methane. Non-methanotrophic methylotrophs and facultative aerobic methanotrophs are more abundant (up to 15.4% of amplicons), but do not correlate with either dissolved gas. *Methylococcaceae* correlate better than all other families in the methane-oxidizing community with the first-order MOx rate constants obtained from the *in situ* incubation data. Changes in the methane-oxidizing community across incubations were inconsistent between experiments but replicable within parallel incubations. The lack of response of the methanotrophic community to ammonium and organic carbon additions suggest these are not limiting.

**Discussion:**

Our results suggest *Methylococcaceae* primarily drive MOx in Jordan lake, despite often not being the most abundant methanotrophic group, and that high oxygen concentrations may suppress this group independently of their association with lower methane concentrations.

## 1 Introduction

Methane is a powerful greenhouse gas, estimated to have up to 32 times the global warming potential—or ability to retain atmospheric heat—as carbon dioxide over a hundred year period (Etminan et al., 2016). About 35%−50% of methane entering the atmosphere comes from natural sources, like wetlands and lakes (Saunois et al., 2020). Aerobic methanotrophs, bacteria that oxidize methane into methanol to generate cellular energy and synthesize biomass, are critical methane sinks, reducing the amount of methane escaping natural sources (Bornemann et al., 2016; Taubert et al., 2019; Yu and Chistoserdova, 2017). Much of what is known about these organisms comes from laboratory studies or static environmental samples, making it difficult to pin down how variations in geochemical factors and aerobic methane-oxidizing communities interact in real time to produce the methane consumption rates observed in nature.

Obligately aerobic methanotrophs; found in the classes *Gammaproteobacteria, Alphaproteobacteria*, and the phylum *Verrucomicrobia* (Knief, 2015); inhabit the oxygenated upper sediment and water column at methane-rich sites (Bornemann et al., 2016; He et al., 2012; Steinle et al., 2015). Using either particulate or soluble methane monooxygenase (pMMO or sMMO), they oxidize methane into methanol and then formaldehyde, from which it is either incorporated into biomass or oxidized for continued energy generation (Hanson and Hanson, 1996). How rapidly these bacteria oxidize methane varies widely by location, with published rates from freshwater lakes ranging from less than 10 nM/day to over 840 μM/day (Donis et al., 2017; Rudd and Hamilton, 1975).

Aerobic methanotrophs may engage in syntrophy with non-methanotrophic methylotrophs, bacteria that consume methylated or single-carbon compounds other than methane, many of which are from the family *Methylophilaceae* (Yu and Chistoserdova, 2017). These two groups of organisms frequently co-occur in natural systems, carbon transfers rapidly from aerobic methanotrophs to non-methanotrophic methylotrophs in *ex situ* incubations, and aerobic methanotrophs undergo transcriptional changes in the presence of non-methanotrophic methylotrophs to facilitate that transfer of carbon (Taubert et al., 2019; Radajewski et al., 2000; Krause et al., 2017). This interspecies carbon transfer likely benefits aerobic methanotrophs, given that the presence of non-methanotrophic methylotrophs can increase the rate of methane removal by a co-cultured population of aerobic methanotrophs, though the exact underlying mechanism is unknown (Jeong and Kim, 2019).

Due to the technological limitations of quantifying methane concentrations *in situ*, little is known about the interactions between *in situ* aerobic methane oxidation (MOx) rates, aerobic methanotrophs, and other geochemical factors. Instead, most results relating to these biogeochemical interactions come from a combination of *ex situ* incubations and static natural measurements (Steinle et al., 2017; Reis et al., 2020; Bornemann et al., 2016). To understand how natural methane-oxidizing communities consume methane, they must be studied *in situ* where they have access to typical concentrations of key parameters (e.g., oxygen, methane, nutrients, etc.), are exposed to natural temporal fluctuations in temperature, salinity, and pH, are surrounded by a diverse biological community, and are minimally disturbed by the incubation/collection process.

Previously, our team developed a novel *in situ* incubation system, called the “iBag system,” that can store and continuously circulate 22 L of ambient water and measure aqueous methane and oxygen concentrations near continuously, all while preventing environmental gas exchange and light penetration (Hudspeth et al., 2024a). Methane dynamics are obtained by fitting the data to the following equation:


$$\begin{array}{l} {\left[CH_{4}\right]}_{t}={\left[CH_{4}\right]}_{initial}e^{-kt} \end{array}$$

where [CH_4_]_t_ is the instantaneous methane concentration (nM) at a given time, which decreases exponentially over the multi-day *in situ* incubation, [CH_4_]_initial_ is the initial methane concentration (nM) after correcting for an initial baseline offset, t is time (h), and *k* is the modeled first-order rate constant (h^−1^). Instantaneous rates of methane oxidation can be calculated by [CH_4_]_t_ multiplied by *k*. Since *k* is the potential of the microbial community in each incubation to oxidize methane, we hypothesized it could be used to determine the roles of different microbes in the community since it represents the summative effect of the abundance, type, and metabolic activity level of the methanotrophs present in each incubation. For instance, if the *k* values do not correlate with the abundance of methanotrophs, this could mean that *k* is driven by variations in cellular metabolic activity or by different methanotroph groups at different times. While the bacteria capable of methanotrophy are well-established in freshwater lakes, it is unclear whether variations in their abundance, their metabolic activity, or their syntrophic partners are more important for determining MOx *in situ*.

Here, we deployed iBag systems in Jordan Lake, NC, six times across 13 months to investigate the interactions between the native aerobic-methane oxidizing community, the natural fluctuations in geochemical and physical parameters, and the resulting first-order rate constant (*k*) of MOx to determine whether abundance, activity, or composition of the methane-oxidizing community best account for the methane oxidizing potential of the lake.

## 2 Materials and methods

### 2.1 Study site

All incubations were performed at Crosswinds Boating Center, Jordan Lake, NC, USA (35° 44′ 36.9″ N 79° 00′ 28.8″ W). Jordan Lake is a man-made, seasonally stratified, eutrophic, freshwater lake. Multiple iBag *in situ* incubation systems were suspended at 3.5 m water depth off of the Center's dock, where water depth was approximately 4.0 m. This depth was chosen to avoid sediment disturbance while maximizing ambient methane concentrations. The first set of incubations was performed in October 2020, three more incubation sets between June and July 2021, and two more during October and November 2021, for a total of six sets of incubations (Table 1).

**Table 1:** Experimental set-up summary.

**Experiment**	**Duration (hours)**	**Sampling timepoints (hours)**	**Replicates**	**Additions**	**Initial addition concentration**
October 2020	50	0, 1, 2, 5, 8, 21, 32, and 50	3	None	N/A
June 2021 - 1	70	0, 3, 7, 15, 24, 32, 40, 63, and 70	3 (1 control, 2 with additions)	Ammonium	20.5 and 21.9 μM
June 2021 - 2	54	0, 4, 7, 10, 19, 31, 44, and 54	3 (1 control, 2 with additions)	Ammonium	43.4 and 45.2 μM
July 2021	45	0, 3, 6, 19, 32, and 45	2 (1 control, 1 with additions)	Ammonium	38.4 μM
October 2021	61	0, 24, 39, and 61	3 (All with additions at ~39 and ~48 h)	Dried algae powder	0.07 g/L (~39 h), 0.33 g/L (~48 h)
November 2021	18	0, 5, 9, and 18	2 (Both with additions)	Dried algae powder	0.401 g/L

Unless otherwise noted, additions were performed at 0 h.

### 2.2 *In situ* incubation system

The iBag system is described in detail in Hudspeth et al. (2024a). Briefly, it consists of a 22 L opaque mylar gas-tight incubation bag, connected via polyethylene tubing and a pump to an Aanderaa (AADI) Series 4430 oxygen optode and a Franatech GmbH Laser Methane sensor, which each had a precision of ±2 μM and ±2 nM, respectively. After flushing ambient lake water through the system for at least an hour until recorded values stabilize, the system is shut off from the outside water. No biofilm formation was observed, but, as with every incubation experiment, the influence of invisible biofilms is possible. Oxygen and methane concentrations are measured continuously in the recirculating water for 18–70 h until oxygen is depleted. The first-order rate constant values for MOx were calculated from exponential fits of the methane concentrations over time (Hudspeth et al., 2024a). Data for the time zero (T0) populations of each incubation analyzed in this paper are listed in Supplementary Table S1. The geochemical data for all experiments, except the November 2021 experiment (Supplementary Figure S1), are reported in Hudspeth et al. (2024a).

### 2.3 Biological sample collection and treatments

Biological samples were taken by attaching a 60 ml BD Luer-Lok™ sterile syringe to a three-way stopcock valve at the end of the sampling port. Two replicate syringes were taken for each timepoint, rinsing the syringe twice with incubation fluids before the sample was taken. The sample water was filtered through a 0.22 μm Sterivex-GV Pressure filter (Supplementary Figure S2) for DNA extraction. During this process, the systems were lifted to the surface of the water for access to the sampling port but remained submerged for temperature regulation. The DNA samples were immediately frozen and kept so until they were delivered to the University of Tennessee for storage in a −80 °C freezer and DNA was extracted a few months later. A total of 125 biological samples were collected, 16 of which were T0 samples that captured well-mixed, unincubated lake water just as the incubations were closed off from the lake. Incubations were performed as described in Table 1. Ammonium and dried algae powder (GNC earth Genius™ Spirulina) were added to incubations as indicated in Table 1.

### 2.4 DNA Extraction

DNA extractions were performed with a phenol-chloroform extraction modified for use on Sterivex filters (Vetriani et al., 1999; Wright et al., 2009). The samples were freeze-thawed three times before being treated with lysozyme (100 mg/ml), proteinase K (20 mg/ml), and sodium dodecyl sulfate (20%) for chemical cell lysis. DNA was purified with phenol-chloroform and then precipitated with sodium acetate (3 M), isopropanol (100%), and ethanol (70%). Tris buffer was used to elute the resulting DNA. DNA concentrations were then measured with a Qubit™ 4 Fluorometer using the Qubit™ 1X High Sensitivity dsDNA Assay kit (Invitrogen, Eugene, OR). Negative controls, consisting of 60 ml of ultrapure water filtered through a new Sterivex, were processed in parallel with the samples. They were prepared either when new extraction reagents were used or when beginning extractions from a new sample set.

### 2.5 16S rRNA gene amplicon processing and analysis

The Illumina MiSeq platform was used to generate amplicons of the V4 region of the 16S rRNA gene with primers 515f (GTGYCAGCMGCCGCGGTAA) and 806R (GGACTACNVGGGTWTCTAAT) (Caporaso et al., 2011; Parada et al., 2016; Apprill et al., 2015) at the University of Tennessee Genomics Core in Knoxville, TN. The dada2 pipeline was used to process the resulting reads into amplicon sequence variants (ASVs) and to then classify them using the SILVA reference set 138 (Callahan et al., 2016; Quast et al., 2013). All chloroplast, mitochondria, and added algae (*Arthrospira* PCC-7345) reads were removed from the overall dataset before analysis. Library sizes per sample post-processing ranged from 36,256 to 207,552 reads and yielded 15,545 ASVs overall. ASVs were normalized to total library size to obtain the relative abundance. A parallel analysis was conducted with rarified samples (randomly selecting a subset of reads so all libraries are the same size before normalizing to library size) and found large and statistically significant correlations between the rarified and the non-rarified datasets (Supplementary Tables S2, S3), indicating that proceeding with the rarefied dataset would provide minimal benefit over proceeding with the original dataset. ASVs taxonomically classified at the family level or lower were assigned to different functional guilds by assessing the range of functional guilds present in a given ASV's family or genus. Aerobic methanotroph and non-methanotrophic methylotroph ASVs were separated by type and then clustered by family. Taxonomic classifications were confirmed by checking the established literature; since *Methylocystaceae* and *Beijerinckiaceae* were collapsed together by the SILVA reference set 138 (Quast et al., 2013), sequences from genera established to belong to *Methylocystaceae* (*Methylocystis* and *Methylosinus*) were manually separated from the rest of the *Beijerinckiaceae* sequences. Taxonomic groups of interest were excluded from further analysis if over 15% of their ASVs were also found in the negative controls or were absent in over 90% of samples overall.

### 2.6 Quantitative PCR

A QuantiNova™ SYBR Green^®^ PCR kit was used to perform quantitative PCR (qPCR). Supplementary Table S4 lists the targeted sequences and primers (Sharp et al., 2012; Kolb et al., 2003; Kubo et al., 2012). qPCR was performed using a BioRad DNA Engine Opticon^®^ 2 Real-Time PCR Detector, with an initial denaturation step of 95 °C for 2 min and 2-step cycling for 35 cycles of 5 s of denaturation at 95 °C and 10 s of annealing and extension at 60 °C. RNAse-free water included with the kit was used as negative controls. Positive controls were either synthesized genes purchased from Eurofins Genomics or amplified to ng/μl concentrations from Jordan Lake water. Melt curve analysis was performed and samples exhibiting primer dimers were considered to have not amplified (C_*t*_ > 35 cycles).

### 2.7 Data analysis

All 16S rRNA gene amplicon relative abundance data and qPCR-measured relative abundance data (family-specific *pmoA* gene copies divided by bacterial 16S rRNA gene copies) were log_10_-transformed before statistical analysis to meet assumptions of normality of errors. Statistical analysis and graphical representation of the population dynamics of the microbial groups of interest and the geochemical variables were performed in R version 4.0.3 (R Core Team, 2020) with RStudio (R Studio Team, 2020).

Complete R code containing all the steps to reproduce our 16S rRNA gene amplicon processing and analysis, model generation and selection, and statistical analysis along with the microbiological and geochemical data analyzed in this study are available at GitHub at https://github.com/JBaily/Jordan_Lake_20-21. The raw geochemical data and the code used to process it can be found at GitHub at https://github.com/zhudspeth/JordanLakePaper (Hudspeth et al., 2024a,b).

### 2.8 Correlations

All correlations were Pearson's product-moment correlations. Samples with missing data for one or more geochemical variable were excluded from analysis. Two-tailed *t*-tests were performed to determine the significance of the correlation coefficient estimates.

### 2.9 Multiple regression

Samples with missing data for one or more variables were excluded from analysis. Predictors were assessed for collinearity prior to analysis by calculating the variance inflation factor (VIF) for a model of random, simulated normal data with all three predictors variables. Since the VIF was never above 10 and |*r*| ≤ 0.7, for any pair of variables, no geochemical variables were excluded from analysis (Dormann et al., 2013). Model selection was performed manually from a pool of models that accounted for all possible combinations of predictors and allowed for interactions between any two predictors in the model (two-way interactions). Three-way interactions were not permitted. “Hinged” interactions, two or more two-way interactions that share a variable (i.e., an oxygen-temperature interaction and an oxygen-methane interaction in the same model), were also excluded. The best models had the lowest corrected Akaike Information Criterion (AICc) and Bayesian Information Criterion (BIC) scores. Model residuals were visually assessed for normality. Models within ~2 AICc and BIC units of the best model were assessed to determine if they explained substantially more variation (adjusted *R*^2^ higher than that of the best model by >= 0.05) than the best model. Adjusted *R*^2^, which reduces the bias introduced by having different numbers of predictors, is used instead of *R*^2^ (Westfall and Arias, 2020).

### 2.10 Population dynamics

The shifts in 16S rRNA gene amplicon relative abundance of the groups of interest during the *in situ* incubations were plotted vs. experimental duration and assessed qualitatively. Statistical differences between the means of the T0 relative abundance data were assessed by performing a Tukey's HSD test on a one-way ANOVA.

### 2.11 Software

R Packages used for statistical analysis were: fBasics (Wuertz et al., 2020), car (Fox and Weisenberg, 2019), olsrr (Hebbali, 2020), survey (Lumley, 2023), interactions (Long, 2019), jtools (Long, 2022), tidyverse (Wickham et al., 2019), dplyr (Wickham et al., 2023), and multcompView (Graves et al., 2023). R Packages used for processing the 16S rRNA gene amplicon reads were: dada2 (Callahan et al., 2016), phyloseq (McMurdie and Holmes, 2013), Biostrings (Pagès et al., 2020), microbiome (Lahti and Shetty, 2019), rioja (Juggins, 2022), and microbiomeutilities (Shetty and Lahti, 2021).

## 3 Results

### 3.1 Aerobic methane-oxidizing community composition during consumption

Methane was consumed in each incubation at a first-order exponential rate with respect to methane concentration, with oxygen concentrations not impacting the observed rates (Hudspeth et al., 2024a). Consumption ceased either when no more methane was left in the system or when the iBag system became anoxic. Oxygen concentrations decreased linearly, except when dried algae was added, and no experiments saw increases in oxygen concentrations. In the two cases where dried algae powder was added, the rate of oxygen consumption, but not that of methane consumption, increased exponentially post-addition (Supplementary Figure S1).

**Figure 1:**
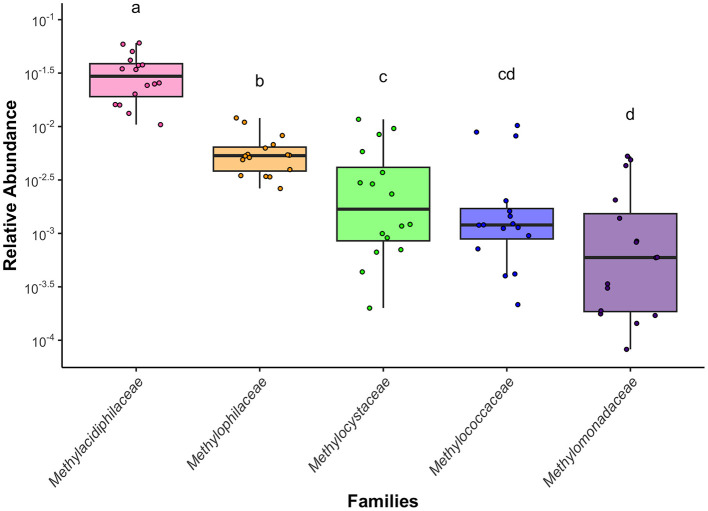
A comparison of the mean 16S rRNA gene amplicon relative abundance of aerobic methanotrophs and non-methanotrophic methylotrophs in Jordan Lake by family. Statistical differences between the relative abundance means were assessed by performing a Tukey's HSD test on a one-way ANOVA. Boxplots show mean, ±SE, and range.

Aerobic methanotrophs and non-methanotrophic methylotrophs from the following families are present in all T0 samples and persist throughout the incubations: *Methylacidiphilaceae, Methylophilaceae, Methylocystaceae, Methylococcaceae*, and *Methylomonadaceae* (Tukey's HSD test on a one-way ANOVA; Figure 1, Supplementary Tables S5, S6). Cultured genera within *Methylococcaceae, Methylomonadaceae, Methylacidiphilaceae*, and *Methylocystaceae* are aerobic methanotrophs (Knief, 2015) and cultured genera of *Methylophilaceae* are non-methanotrophic methylotrophs (Salcher et al., 2015). The dominant genera from *Methylococcaceae* and *Methylomonadaceae* in this system, which are *Methyloparacoccus* and *Methylomonas*, respectively, are obligate methanotrophs (Vekeman, 2019; Bowman, 2014). *Methylocystis*, the dominant genus from *Methylocystaceae* in Jordan lake, is known to contain several facultative species (Haque et al., 2020). However, no *Methylacidiphilaceae* 16S rRNA sequences could be identified past the family level, indicating *Methylacidiphilaceae* in Jordan Lake may display different physiology than the previously cultured genera from this family, which are thermophilic aerobic methanotrophs (Knief, 2015).

### 3.2 Assessing potential for methanotrophy with *pmoA* genes

We surveyed for the presence of pMMO subunit A (*pmoA*) genes from the families of *Methylococcaceae* and *Methylacidiphilaceae* in all T0 samples using qPCR (Supplementary Table S4) (Sharp et al., 2012; Kolb et al., 2003). *Methylococcaceae pmoA* amplified in all incubations except November 2021 and one of the October 2021 incubations. *Methylacidiphilaceae pmoA* primers did not amplify in any sample, although the positive controls for those primers worked.

### 3.3 Assessing quantitativeness of 16S rRNA gene amplicon relative abundance

**Figure 2:**
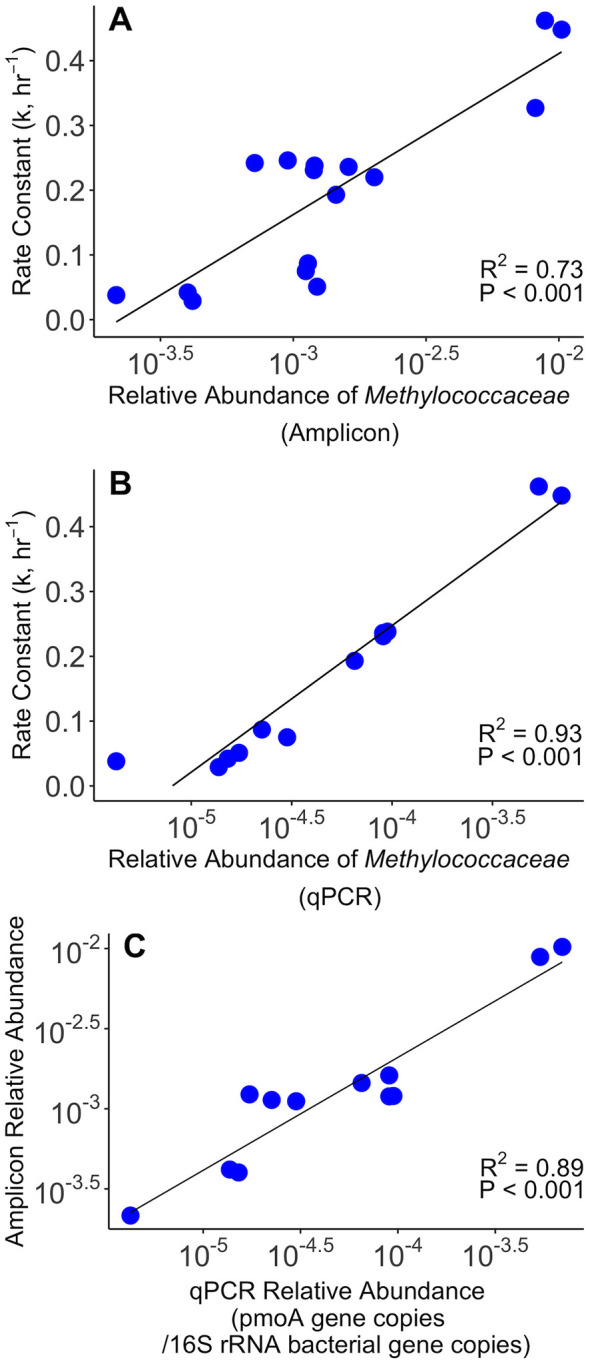
Correlations between the aerobic methane oxidation (MOx) first-order rate constant, 16S rRNA gene amplicon- and the qPCR-measured relative abundances of *Methylococcaceae*. Correlation between the 16S rRNA gene amplicon relative abundance and the first-order rate constant (*R*^2^ = 0.73, *t*(14) = 6.20, *P* < 0.001) **(A)**, between the qPCR relative abundance of *Methylococcaceae* (*Methylococcaceae pmoA* gene copies/bacterial 16S rRNA gene copies) and the first-order rate constant (*R*^2^ = 0.93, *t*(10) = 11.69, *P* < 0.001) **(B)**, and between the 16S rRNA gene amplicon and qPCR relative abundances of *Methylococcaceae* (*R*^2^ = 0.89, *t*(10) = 8.95, *P* < 0.001) **(C)**. All are Pearson's product-moment correlations.

To determine if primer bias skewed the relative amounts of taxa across different 16S rRNA gene amplicon datasets, we compared the amplicon- and qPCR-measured (family-specific *pmoA* gene copies/bacterial 16S rRNA gene copies) relative abundance of *Methylococcaceae*. The amplicon- and qPCR-measured relative abundances of *Methylococcaceae* exhibit a strong, positive correlation (*R*^2^ = 0.89, *t*(10) = 8.95, *P* < 0.001) across all T0 populations (Figure 2C). Amplicon- and qPCR-measured relative abundances correlate positively across all the October 2020 incubations samples (*R*^2^ = 0.41, t(13) = 3.02, *P* = 0.010; Supplementary Figure S3), exhibiting qualitatively similar shifts (e.g., both increasing at the same time) even if the magnitude differed. As these correlations indicate that the background population does not substantially alter primer bias across the T0 population samples, this principle can be extended to all other taxa in the community (Lloyd et al., 2020; McLaren et al., 2022).

### 3.4 Correlations of T0 populations of methylotrophs and methanotrophs with geochemistry

Out of all of the families of methanotrophs and non-methanotrophic methylotrophs, the 16S rRNA gene amplicon relative abundances of *Methylococcaceae* from the T0 populations exhibit the strongest positive correlation with the first-order rate constant of MOx (*R*^2^ = 0.73, *t*(14) = 6.20, *P* < 0.001; Figure 2A, Supplementary Table S7). The qPCR relative abundance of *Methylococcaceae* also correlates strongly with the first-order rate constant of MOx (*R*^2^ = 0.93, *t*(10) = 11.69, *P* < 0.001; Figure 2B, Supplementary Table S8). This family consists of obligate aerobic methanotrophs and primarily consists of the genus *Methyloparacoccus* in Jordan Lake. The 16S rRNA gene amplicon relative abundance of the genus *Methyloparacoccus* alone correlates even more strongly with the first-order rate constant of MOx (*R*^2^ = 0.78, *t*(14) = 7.02, *P* < 0.001) than the family as a whole does. These correlations with *Methylococcaceae* and *Methyloparacoccus* are the strongest out of all correlations between the first-order rate constant of MOx and the families and genera present in all T0 populations (Supplementary Tables S9, S10). While the next strongest positive correlation between a family and the first-order rate constant is similar in strength to the correlation with *Methylococcaceae* (*Neisseriaceae*: *R*^2^ = 0.72), the next strongest positive correlation between a genus and the first-order rate constant is considerably weaker (*Prochlorothrix*: *R*^2^ = 0.60). *Prochlorothrix* does not belong to *Neisseriaceae*, suggesting the correlation with *Neisseriaceae* is spurious. All other methanotrophic and methylotrophic families, except *Methylophilaceae*, correlate less strongly with the first-order rate constant (Supplementary Table S7). *Methylophilaceae*, in contrast, does not correlate at all.

Both *Methylococcaceae* and *Methyloparacoccus* correlate strongly with the initial rate of MOx (Supplementary Tables S11, S12). However, this correlation with the initial rate is not only weaker than the correlation with the rate constant in both cases (*Methylococcaceae*: *R*^2^ = 0.65, *t*(14) = 5.15, *P* < 0.001; *Methyloparacoccus*: *R*^2^ = 0.71, *t*(14) = 5.80, *P* < 0.001), but two families (*Isosphaeraceae* and cvE6) correlate more strongly with the initial MOx rate than *Methylococcaceae* does (Supplementary Table S11). The gap in strength between the correlation with the initial rate and *Methyloparacoccus* and the next most significant genus is also over two times smaller than the gap associated with the rate constant (Initial rate: *R*^2^ = 0.71 vs. *R*^2^ = 0.64; Rate constant: *R*^2^ = 0.78 vs. *R*^2^ = 0.60; Supplementary Tables S10, S12).

**Figure 3:**
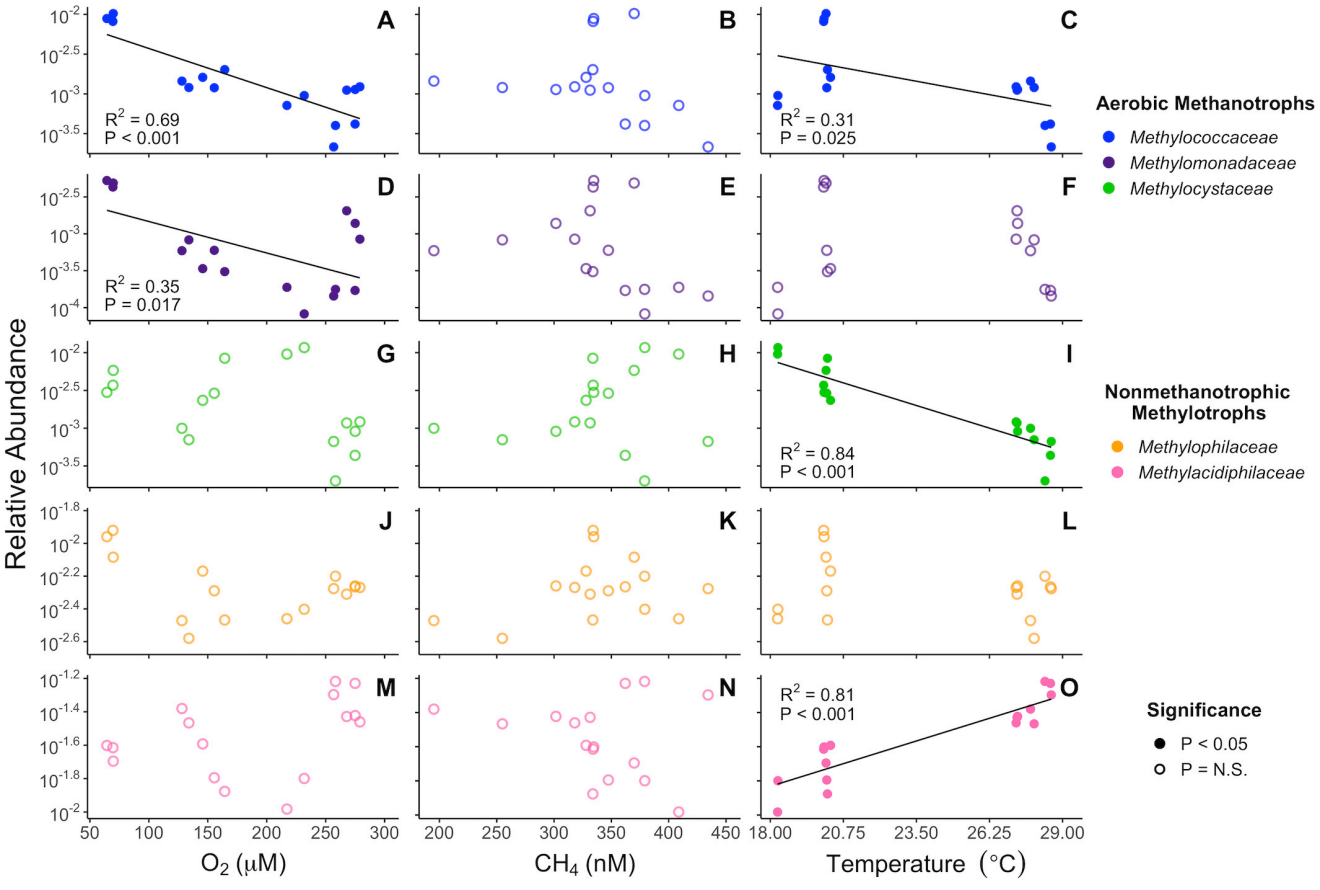
T0 relative 16S rRNA gene amplicon abundance of methanotrophic and methylotrophic families correlated with initial geochemical parameters. Correlations with *Methylococcaceae* **(A, B, C)**, *Methylomonadaceae* **(D, E, F)**, *Methylocystaceae* **(G, H, I)**, *Methylophilaceae* **(J, K, L)**, and *Methylacidiphilaceae* **(M, N, O)** are represented. Geochemical parameters are initial oxygen concentration **(A, D, G, J, M)**, initial methane concentration **(B, E, H, K, N)**, and initial temperature **(C, F, I, L, O)**. Most families significantly correlate with either temperature or oxygen, but none significantly correlate with methane. Trendlines are only present for significant correlations. All are Pearson's product-moment correlations. Correlations are listed in Supplementary Table S13.

Only the families of obligate aerobic methanotrophs, *Methylococcaceae* (*R*^2^ = 0.69, *t* = −5.52, and *P* < 0.001) and *Methylomonadaceae* (*R*^2^ = 0.35, *t* = −2.72, and *P* = 0.017), correlate with oxygen, both inversely (Figure 3). *Methylococcaceae* and *Methylocystaceae*, facultative aerobic methanotrophs, correlate inversely with temperature (*R*^2^ = 0.31, *t* = −2.51, and *P* = 0.025; *R*^2^ = 0.84, *t* = −8.43, and *P* < 0.001). The qPCR relative abundances of *Methylococcaceae* correlate similarly: inversely with oxygen, inversely with temperature, and not at all with methane (Supplementary Table S8). *Methylacidiphilaceae*, presumed non-methanotrophic methylotrophs, correlate positively with temperature (*R*^2^ = 0.81, *t* = 7.81, and *P* < 0.001). The other non-methanotrophic methylotrophs from *Methylophilaceae* do not significantly correlate with either of the other two geochemical variables (Oxygen: *t* = −1.49 and *P* = 0.16. Temperature: *t* = −0.933 and *P* = 0.37). None of the five methanotrophic or methylotrophic families significantly correlate with the methane. All amplicon-geochemistry correlations are listed in Supplementary Table S13 and Figure 3.

To assess whether these shifts in *Methylococcaceae* relative abundance reflect actual *Methylococcaceae* population growth or are byproducts of the background bacterial population decreasing in response to low oxygen concentrations, *Methylococcaceae-*specific *pmoA* gene copies and bacterial 16S rRNA gene copies in the T0 samples were correlated with oxygen concentration, methane concentration, and the first-order rate constant of MOx. *Methylococcaceae-*specific *pmoA* gene copies correlate inversely with oxygen (*R*^2^ = 0.41, *t*(10) = −2.63, and *P* = 0.025) and positively with the first-order rate constant of MOx (*R*^2^ = 0.41, *t*(10) = 2.63, and *P* = 0.025). Bacterial 16S rRNA gene copies instead correlate positively with oxygen (*R*^2^ = 0.55, *t*(12) = 3.87, and *P* = 0.002) and inversely with the first-order rate constant of MOx (*R*^2^ = 0.67, *t*(12) = −4.97, and *P* < 0.001). Neither significantly correlates with methane.

### 3.5 Combined effects of oxygen, methane, and temperature

To investigate which correlations are significant when the effect of multiple variables is considered, linear models were constructed via multiple regression for the 16S rRNA gene amplicon relative abundance of each of the five methanotrophic and methylotrophic families in the T0 populations (Supplementary Tables S14, S15). The best models for the obligate aerobic methanotrophs, *Methylococcaceae* (Adjusted *R*^2^ = 0.89, *F*(3,12) = 42.08, *P* < 0.001) and *Methylomonadaceae* (Adjusted *R*^2^ = 0.73, *F*(4,11) = 42.08, *P* < 0.001), contain the predictors of methane concentration, oxygen concentration, and the two-way interaction between those predictors, with temperature as well for *Methylomonadaceae*. Temperature is the sole significant predictor for *Methylocystaceae* (Adjusted *R*^2^ = 0.84, *F*(1,14) = 71.10, *P* < 0.001) and has a negative effect on the relative abundance. Both *Methylophilaceae* (Adjusted *R*^2^ = 0.81, *F*(2,13) = 42.08, *P* < 0.001) and *Methylacidiphilaceae* (Adjusted *R*^2^ = 0.85, *F*(3,12) = 30.01, *P* < 0.001), the non-methanotrophic methylotrophs, have best models that contain oxygen concentration, temperature, and the two-way interaction between those two predictors (Supplementary Figure S4, Supplementary Table S16). None of the geochemical predictors exhibited collinearity (Supplementary Table S17).

**Figure 4:**
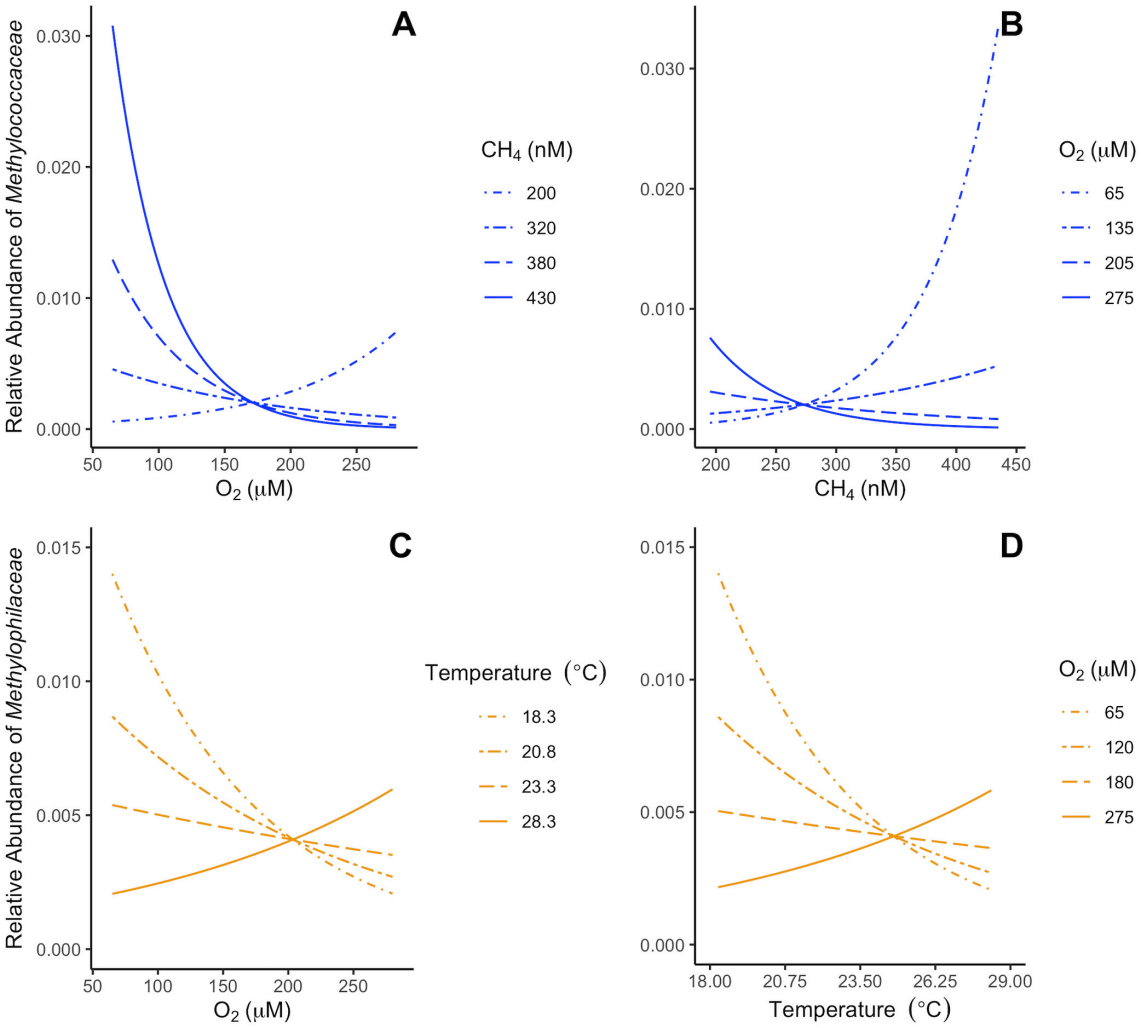
Modeled effects of continuous-by-continuous two-way interactions on the 16S rRNA gene amplicon abundance of *Methylococcaceae* and *Methylophilaceae*. The predicted effects of oxygen concentration **(A)** and methane concentration **(B)** on *Methylococcaceae* modulated by the other variable. The predicted effects of oxygen concentration **(C)** and temperature **(D)** on *Methylophilaceae* modulated by the other variable. Each line represents the effect of the *x* axis variable when the reciprocal variable is held constant at one of four values for which the effect is significantly different from zero.

The interaction between the relative abundance of both *Methylococcaceae* and *Methylomonadaceae* and the concentrations of oxygen and methane is predicted to change slightly depending on the concentration of the other dissolved gas (Figures 4A, B). Oxygen is predicted to have strong negative effect on *Methylococcaceae* and *Methylomonadaceae* when methane concentrations are higher. As methane concentrations diminish, this negative effect of oxygen on relative abundance is predicted to weaken until oxygen is predicted to have a positive effect when methane concentrations on the lower end of what was observed. Due to few data points in this concentration range, the predicted positive effect of oxygen is not robust. Though methane accounts for much less variation than oxygen does, it is predicted to have a positive effect on the relative abundance when oxygen is lower and a negative effect when it is higher (Supplementary Figures S5A, B). The combined effects of oxygen and methane suggests that obligate aerobic methanotrophs are most abundant in environments with low oxygen and high methane concentrations (Figures 4A, B).

Oxygen and temperature interact to influence the relative abundance of *Methylophilaceae* and *Methylacidiphilaceae*. For *Methylophilaceae*, temperature is predicted to have a negative effect when the oxygen concentration are low that becomes positive as oxygen increases (Figures 4C, D), similarly oxygen is predicted to have a negative effect at low temperatures and a positive effect as they increase. For *Methylacidiphilaceae*, temperature is never predicted to have a negative effect on the relative abundance regardless of the oxygen concentration but is predicted to have a positive effect for most of the oxygen concentrations observed (Supplementary Figure S5C). Oxygen is predicted to have a negative effect on its relative abundance only at lower temperatures.

### 3.6 Growth dynamics during aerobic methane oxidation

**Figure 5:**
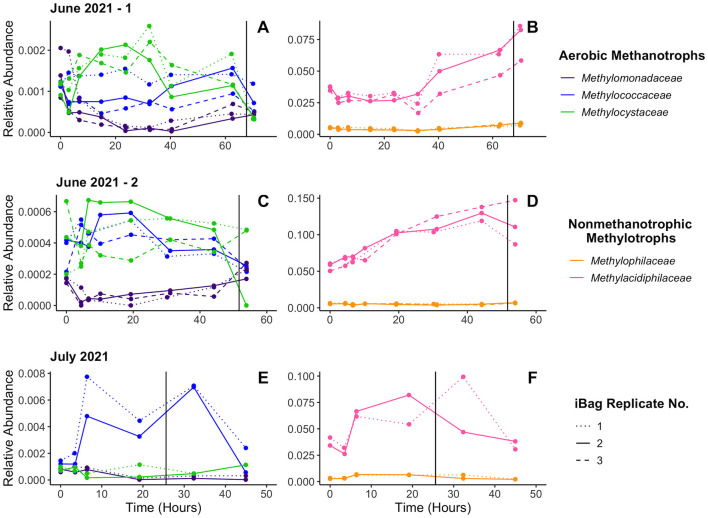
16S rRNA gene amplicon relative abundance data from the summer incubations. Three sets of *in situ* incubations were performed during the Summer months: one in June 2021 **(A, B)**, a second in June 2021 **(C, D)**, and one in July 2021 **(E, F)**. Incubations ended shortly after becoming anoxic. Vertical lines indicate when oxygen concentrations fell below 20 μM. Immediately after starting the incubations, ammonium was added to the first and third iBags deployed, with the second serving as a control. The ammonium concentrations of the iBags that received ammonium in the first June 2021 experiment rose to ~21 μM and those that did in the other two experiments rose to ~42 μM. The 16S rRNA gene amplicon relative abundance of aerobic methanotrophs **(A, C, E)** and the non-methanotrophic methylotrophs **(B, D, F)** by family are shown.

**Figure 6:**
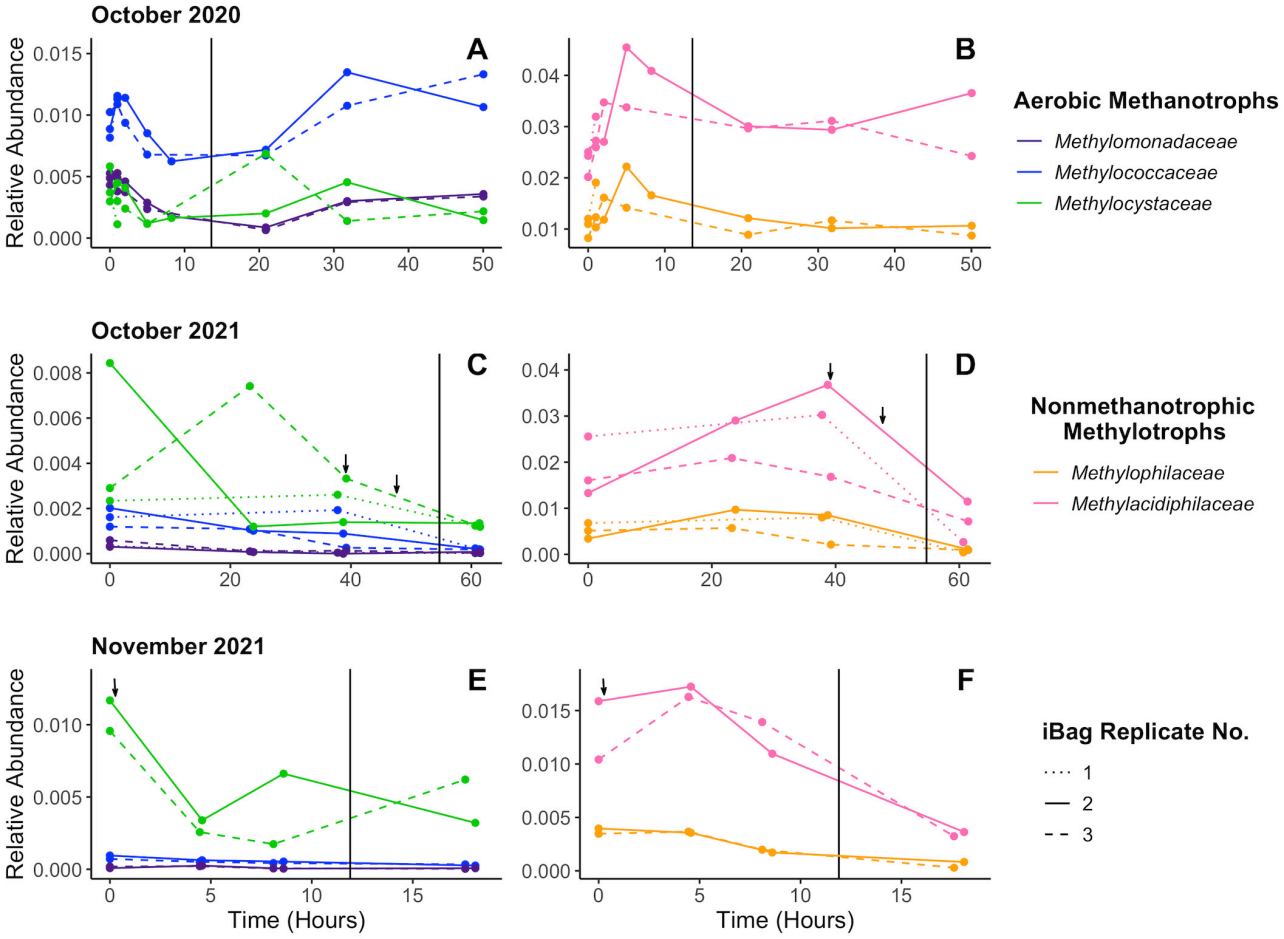
16S rRNA gene amplicon relative abundance data from the fall incubations. Three sets of *in situ* incubations were performed during the Fall months: one in October 2020 **(A, B)**, one in October 2021 **(C, D)**, and one in November 2021 **(E, F)**. Incubations ended shortly after becoming anoxic. Vertical lines indicate when oxygen concentrations fell to 20 μM. The October 2020 experiment was unamended; dried algae was added to all replicates of the October 2021 experiment at ~39.16 and 47.61 h, at initial concentrations of 0.07 and 0.33 g/L, respectively; and dried algae was also added to the November experiment at ~0.275 h, at initial concentrations of 0.40 g/L. Additions are indicated by the arrows. The 16S rRNA gene amplicon relative abundance of aerobic methanotrophs **(A, C, E)** and the non-methanotrophic methylotrophs **(B, D, F)** by family are shown.

All five methanotrophic and non-methanotrophic methylotrophic families had 16S rRNA gene amplicon exhibited consistent shifts in abundance during replicate incubations in both the summer (Figure 5) and fall (Figure 6). These shifts occurred on time scales long enough that they could be attributed to population growth instead of just cell death, as related aerobic methanotrophs and non-methanotrophic methylotrophs exhibit doubling times between 1.5 and 7 h (Bedekar et al., 2023; Bordel et al., 2019; Batey et al., 1996). Despite large differences in overall abundance during the summer, the non-methanotrophic methylotrophs continue exhibit similar abundance shifts in replicate incubations (Supplementary Figure S6). *Methylophilaceae* and *Methylacidiphilaceae*, both likely non-methanotrophic methylotrophs, initially grow and then decline when the incubation water becomes hypoxic. Aerobic methanotrophs tend to either initially decline or maintain a stable population size. Non-methanotrophic methylotrophs are more abundant on average than the aerobic methanotrophs in the T0 populations (Figure 1) and the gap between them tends to increase over time. *Methylacidiphilaceae* is almost always the most abundant, reaching up to 15% of the total prokaryotic population in one incubation (Figure 5D).

Ammonium (~21 or ~42 μM) and dried algae (8.43 ± 0.02 g) were added to some of the summer and fall incubations, respectively. Though the addition of the dried algae powder accelerated oxygen respiration, neither of the additions had any noticeable effect on the population dynamics of any of the families of interest or MOx rates.

## 4 Discussion

We investigated whether the abundance of each member of the native aerobic-methane oxidizing community in a eutrophic lake correlates to the *in situ* first-order rate constant (*k*) of MOx. The lake contained typical communities of obligate aerobic methanotrophs (*Methylococcaceae*, mostly from the genus *Methyloparacoccus*, and *Methylomonadaceae*, mostly from the genus *Methylomonas*) (Knief, 2015; Bowman, 2014), facultative aerobic methanotrophs (*Methylocystaceae*, mostly from the genus *Methylocystis*) (Haque et al., 2020), and non-methanotrophic methylotrophs (*Methylophilaceae*, with “*Candidatus Methylopumilus*”— obligate methylotrophs—being the most abundant genus) (Salcher et al., 2015). *Methylophilaceae* are the only family of non-methanotrophic methylotrophs known to engage in potential syntrophy with aerobic methanotrophs (Yu and Chistoserdova, 2017; Krause et al., 2017; Taubert et al., 2019), suggesting the potential for syntrophic interactions to influence MOx in this system.

*Methylacidiphilaceae*, whose cultured genera are thermophilic aerobic methanotrophs (Knief, 2015), could not be identified past the family level, suggesting the *Methylacidiphilaceae* inhabiting Jordan Lake may have a different physiology from the cultured genera. We assessed their potential for aerobic methanotrophy by amplifying *pmoA* with primers specific to *Methylacidiphilaceae* (Sharp et al., 2012). No samples amplified, even though the primer sets designed for the *Methylococcaceae pmoA* amplified. Since *pmoA* encodes a critical subunit of pMMO and cultured *Methylacidiphilaceae* have not been reported to encode the rarer sMMO (Sharp et al., 2012; Kruse et al., 2019), we conclude that it is unlikely that the *Methylacidiphilaceae* in Jordan Lake oxidize methane and are likely non-methanotrophic methylotrophs instead. This conclusion aligns with reports of mesophilic, uncultured genera closely related to *Methylacidiphilaceae* that lack *pmoA* (Sharp et al., 2014).

### 4.1 Predicted drivers of aerobic methane oxidation

All three families of aerobic methanotrophs in the T0 samples correlate to some extent with the first-order rate constant for MOx. The obligate aerobic methanotroph family *Methylococcaceae* correlates the strongest by far out of three families (Figure 2A, Supplementary Table S7, *R*^2^ = 0.73, *P* < 0.001), even though it is frequently not the most abundant. Even when all families and genera are considered (not just those with cultured aerobic methanotrophs), *Methylococcaceae* and *Methyloparacoccus* still exhibit the strongest correlation with the first-order rate constant (Supplementary Tables S9, S10), suggesting these correlations are not spurious and reflect a real relationship between the rate constant and these bacteria.

These results suggest that the abundance of *Methylococcaceae*, rather than changes in its cellular metabolic activity, or its dynamics relative to other microbial taxa, may drive methane consumption in Jordan Lake, even though *Methylococcaceae* is often less abundant than other methanotrophs. Since changes in relative abundance can reflect real shifts in the population size of a target of interest, this relationship suggests that *Methylococcaceae* in Jordan Lake maintains fairly consistent amounts of pMMO per cell, with MOx mostly a product of population size rather than of pMMO per cell. Transcription levels of *pmoCAB* are known to rapidly vary in response to changing copper concentrations (Tentori and Richardson, 2020), but remain steady under a range of oxygen concentrations, methane concentrations, and nitrogen sources (Yu et al., 2017). If this hypothesis is correct, so long as interfering variables such as copper availability are accounted for, MOx rate constants could be roughly estimated from the abundance of the dominant aerobic methanotrophs in other systems.

The potential of *Methylococcaceae* to be the primary drivers of aerobic methanotrophy in Jordan Lake is consistent with results from other environmental studies showing *Methylococcaceae* are both abundant in freshwater lakes (Bornemann et al., 2016; Beck et al., 2013; Rissanen et al., 2021) and associated with high MOx rates (Bornemann et al., 2016; Reis et al., 2020; Taubert et al., 2019; He et al., 2012). Our results further suggest that they could be the major drivers of MOx in freshwater lakes even when not the most abundant methanotrophic group. Minor fluctuations in the rate constant could further be attributed to minor shifts in pMMO synthesis and activity. However, our results are only correlative and thus cannot definitively prove the existence of this interaction. Further work involving direct measurements of activity (e.g., *ex situ* incubations combined with detailed abundance data, transcriptomics, SIP, proteomics, etc.) is needed to verify if population abundance of the dominant aerobic methanotrophs is what truly drives this correlation or if it is instead another underlying mechanism that also correlates with abundance.

### 4.2 Sensitivity of obligate aerobic methanotrophs to oxygen

Given the tight correlation of T0 *Methylococcaceae* with the first-order MOx rate constant, we examined whether these bacteria–along with the rest of the aerobic methane-oxidizing community–correlate with geochemical and physical parameters previously identified as influencing MOx (Hanson and Hanson, 1996). Both families of obligate aerobic methanotrophs exhibit strong inverse correlations with oxygen concentration (Figure 3), driven both by a decrease in absolute abundance of aerobic methanotrophs as well as an increase in the background heterotrophic bacterial population at higher oxygen concentrations, as shown by how the absolute copy numbers of *Methylococcaceae*-specific *pmoA* and bacterial 16S rRNA correlate with oxygen concentrations. The best-fit multiple regression models also suggest that oxygen alone accounts for over half of and a little less than a quarter of all variation in *Methylococcaceae* and *Methylomonadaceae* relative abundance, respectively (Supplementary Table S15). This observation agrees well with previous observations that aerobic methanotrophs can exhibit microaerophilic tendencies both in mixed culture and in the environment (Steinle et al., 2017; Zhu et al., 2017; Rissanen et al., 2021; Reis et al., 2020) and could explain why the first-order rate constant of aerobic methane oxidation exhibits a strong negative correlation with oxygen concentration (Hudspeth et al., 2024a). Gammaproteobacterial aerobic methanotrophs possess adaptations that ensure adequate oxygen reaches their pMMO enzymes under hypoxia (Chen et al., 2012; Weiblen et al., 2025), which is consistent with cells tolerating low oxygen concentrations to access methane originating from anoxic environments (Kuivila et al., 1988). However, in our dataset methane only correlates with aerobic methanotrophs once the negative effect with oxygen is accounted for (Figure 4) and not at all on its own (Figure 3). Other studies have also reported little to no correlation between aerobic methanotroph biomass and methane concentrations (Reis et al., 2020; Samad and Bertilsson, 2017). This suggests that oxygen, more so than methane, may be the direct driver for this trend in aerobic methanotrophs.

There are several possible explanations for why higher oxygen concentrations are detrimental to some aerobic methanotrophs. The first is that oxygen inhibits nitrogenase and could cause nitrogen-limitation that can be alleviated by the addition of over 20 μM ammonium (Rudd et al., 1976). However, we observed no response of aerobic methanotrophs to additions of ammonium above that threshold (~21 or ~42 μM) in the *in situ* incubations (Figure 5), which agrees with these ammonium additions not significantly affecting the MOx rates of these incubations (Hudspeth et al., 2024a). Historically, nitrogen measurements in Jordan Lake are high (mean of 0.93 mg/L) (NC Division of Water Resources, 2021), which is consistent with aerobic methanotrophs not being nitrogen-limited here in Jordan Lake. Finally, the *Methylococcaceae* in Jordan Lake may lack nitrogenase altogether, since cultures of *Methyloparacoccus*, the primary genus in Jordan Lake, do not possess that enzyme (Nguyen et al., 2019). For these reasons, inhibition of nitrogenase seems unlikely as the mechanism for microaerophily. While unlikely, this potential explanation cannot be completely discarded without transcriptomic and/or metabolic data verifying if any members of the aerobic methane-oxidizing community in Jordan Lake fix nitrogen under the observed conditions.

A second explanation could be oxygen toxicity from reactive oxygen species (Steinle et al., 2017), however, cultured species from the dominant genera of *Methylococcaceae* and *Methylomonadaceae* (*Methyloparacoccus* and *Methylomonas*) in Jordan Lake contain catalase and superoxide dismutase, which can may mitigate the effects of reactive oxygen species (Nguyen et al., 2019; Hoefman et al., 2014).

A hypothesis for future study that fits with our observations is that aerobic methanotrophs may accumulate more of their toxic metabolic intermediate formaldehyde under high oxygen conditions, as has been observed *ex situ* (Costa et al., 2001). Formaldehyde is highly cytotoxic, capable of severely damaging both the proteins and DNA of bacteria (Chen et al., 2016), but also an unavoidable intermediate in aerobic methanotrophy (Hanson and Hanson, 1996). This explanation aligns neatly with the possibility that obligate aerobic methanotrophs, at least from the family *Methylococcaceae*, do not substantially regulate their amount of pMMO under these conditions, as suggested by our rate constant correlations (Figure 2). A large standing pool of pMMO would leave aerobic methanotrophs vulnerable to high environmental concentrations of oxygen, especially considering the rapid diffusion of oxygen into the cell (Imlay, 2025).

This microaerophily may also be a result of deficiencies or imbalances of other nutrients not measured in this study, such as copper which is critical for the synthesis of pMMO (Hanson and Hanson, 1996). Regardless of the exact mechanism, it is notable that access to methane may not drive aerobic methanotroph microaerophily under all observed conditions.

### 4.3 Oxygen insensitivity in non-methanotrophic methylotrophs and facultative aerobic methanotrophs

In contrast, we found that neither methane nor oxygen by themselves are significant predictors of the relative abundance of non-methanotrophic methylotrophs and facultative aerobic methanotrophs. Instead, temperature or temperature and oxygen best predict their abundance (Figures 4C, D; Supplementary Table S15), likely because facultative aerobic methanotrophs can consume alternate compounds, avoiding the formaldehyde production associated with MOx key to the formaldehyde hypothesis (Haque et al., 2020). Some non-methanotrophic methylotrophs could also avoid the excess formaldehyde production in the formaldehyde hypothesis through the use of other methylated substrates such as trimethylamine (Martinez-Gomez et al., 2013). Even those that primarily consume methanol would be insulated from this effect as methanol is a polar compound that likely undergoes slower, active transport into the cell (Patra et al., 2006; Agrawal and Flores, 1989). Such regulation may afford non-methanotrophic methylotrophs better control of the methanol oxidation pathway under high oxygen concentrations, thereby avoiding excess formaldehyde production.

### 4.4 Temperature response

The linear models suggest that the inverse correlation of *Methylococcaceae* and temperature is an indirect effect of the correlation between temperature and oxygen. The negative effect of temperature on *Methylocystis* (Figure 4C) is surprising, since cultures of this genus tend to prefer warmer temperatures between 25 and 37 °C (Jung et al., 2020). It is possible that competition with other microbes for food sources during the warmer months manifests as a negative response to temperature (Hudari et al., 2020; van Bodegom et al., 2001). The strong positive correlation between non-methanotrophic methylotrophic *Methylacidiphilaceae* and temperature likely results from increased metabolism at higher temperatures, or the presence of fresher organic matter in spring and summer months.

The other non-methanotrophic methylotroph family *Methylophilaceae* responds to temperature positively when oxygen is high and inversely to temperature when oxygen is low (Figures 4C, D). This finding could result from *Methylophilaceae* not engaging in syntrophy with obligate aerobic methanotrophs in summer months and instead relying on photosynthetically-derived organic matter, allowing its metabolism to increase with the proxy of temperature. In colder months where photosynthetic organic matter is less available (Tonetta and Petrucio, 2020), it may then partner with obligate methanotrophs and therefore follow along with their inverse relationship to temperature.

### 4.5 Incubations

We found that aerobic methanotroph (*Methylococcaceae, Methylomonadaceae*, and *Methylocystaceae*) abundance patterns over the course of *in situ* methane oxidation incubations are replicable within each incubation experiment but change across different experiments (Figures 5, 6). The consistency between replicate incubations within individual experiments indicates that relative abundance changes were not random, but the inconsistency across different experiments suggests that other factors (i.e., viral load, organic matter, etc.) drive the population changes. The lack of consistent response to any set of geochemical conditions (e.g., oxygen concentrations dropping below 20 μM) suggests lowered methane and/or oxygen concentrations for the duration of the incubations (< 75 h) are insufficient to strongly promote population growth or decline. The ability to hold out during temporary “famine-like” periods until methane and/or oxygen is renewed in the environment would benefit aerobic methanotrophs at sites where methane production is inconsistent.

Non-methanotrophic methylotrophs grow until oxygen is nearly consumed at the end of the incubations. Their populations begin shrinking once oxygen concentrations drops below ~20 μM, which aligns with them being obligate aerobes (Doronina et al., 2014). The growth patterns of both families (*Methylophilaceae* and *Methylacidiphilaceae*) closely mirror each other across the replicate incubations of each experiment, with the exception of the July 2021 incubations (Figures 5E, F). The consistency in behavior of both *Methylophilaceae* and *Methylacidiphilaceae* across nearly all experiments suggests that the similar factors act on both groups, supporting our hypothesis that the *Methylacidiphilaceae* found in Jordan Lake are non-methanotrophic methylotrophs too and suggesting that these non-methanotrophic methylotrophs are more sensitive to changing geochemical conditions than the aerobic methanotrophs.

No changes are observed with the addition of either ammonium or dried algae relative to unamended controls. The insensitivity of both groups to ammonium implies that they are either not nitrogen limited or do not use ammonium. The lack of response to dried algae suggests that non-methanotrophic methylotrophs preferentially consume single-carbon compounds over the complex algal necromass. The dried algae does stimulate the broader microbial community, as the rate of oxygen consumption rapidly accelerates when the dried algae is added (Supplementary Figure S1). This consumption trend is also observed in the relative abundance of 16S rRNA gene amplicons associated with the dried algae (*Arthrospira* PCC-7345), which spike immediately post-addition and decrease rapidly thereafter, as would be expected if some microbes are consuming organic matter from the algal necromass (Supplementary Figure S7). Combined, these results support the broader heterotrophic community having little immediate impact on the aerobic methane-oxidizing community.

## 5 Conclusions

We found that the abundance of *Methylococcaceae*, a family of known obligate aerobic methanotrophs, correlates tightly with the first-order rate constant of MOx measured in *in situ* incubations. This suggest that the kinetics of MOx could be primarily controlled by how many active aerobic methanotrophs there are in a microbial population, rather than by the shifts in their metabolic activity. Determining how different aerobic methanotroph taxa contribute to methane oxidation in that case may not be as simple as assessing their comparative abundance, as *Methylococcaceae* are not always the most abundant aerobic methanotroph family in this system. The iBag *in situ* incubation system enabled us to couple precise, real-time geochemical measurements with biological data in order to calculate accurate rate constants for each incubation, permitting us to explore the microbiological capacity for methane consumption at naturally-varying methane and oxygen concentrations.

*Methylophilaceae* are common non-methanotrophic methylotrophs in this microbial community, suggesting that the role of syntrophy in MOx should be further explored in this system. In addition, the members of *Methylacidiphilaceae* observed in Jordan Lake may also be non-methanotrophic methylotrophs, as they exhibit abundance patterns consistent with those of *Methylophilaceae* and lack the *pmoA* gene, even though other genera from this family are aerobic methanotrophs (Knief, 2015).

Curiously, both families of obligate aerobic methanotrophs identified in Jordan Lake exhibit no correlation with methane concentration, instead correlating strongly and inversely with oxygen concentration. Methane concentration further is only a significant predictor of relative abundance when permitted to interact with oxygen, an interaction which suggests these *Methylococcaceae* and *Methylomonadaceae* will only bloom under high methane and low oxygen conditions. Combined, these results indicate that these methanotrophs experience some direct negative effect from higher oxygen concentrations that is unrelated to methane access. By using the iBag *in situ* incubation system we not only are able to link specific microbial groups to MOx rate constants but also shed light on an underexplored aspect of aerobic methanotroph physiology by using the system start to tease apart the independent effects of oxygen and methane concentrations *in situ*.

## Data Availability

The data presented in this study are publicly available. The data can be found here: https://www.ncbi.nlm.nih.gov/, accession PRJNA1117504.
