# Supplementary material for: Linking microbes to in situ methane oxidation rates in a eutrophic freshwater lake
Source: Front Microbiol. 2026 Mar 6;17:1789101. doi: 10.3389/fmicb.2026.1789101 (PMC13002849; doi:10.3389/fmicb.2026.1789101)
Supplement: Supplementary file 1 [file Data_Sheet_1.pdf]

Supplementary Material

For all statistical analysis, amplicon and qPCR derived relative abundances are log_{10}-transformed to meet assumptions of normality or normality of errors. qPCR relative abundance is *pmoA* gene copies from the assessed family / bacterial 16S rRNA gene copies and amplicon relative abundance is the proportion of the 16S rRNA gene amplicon library belonging to that taxonomic group.

Supplemental Tables and Figures

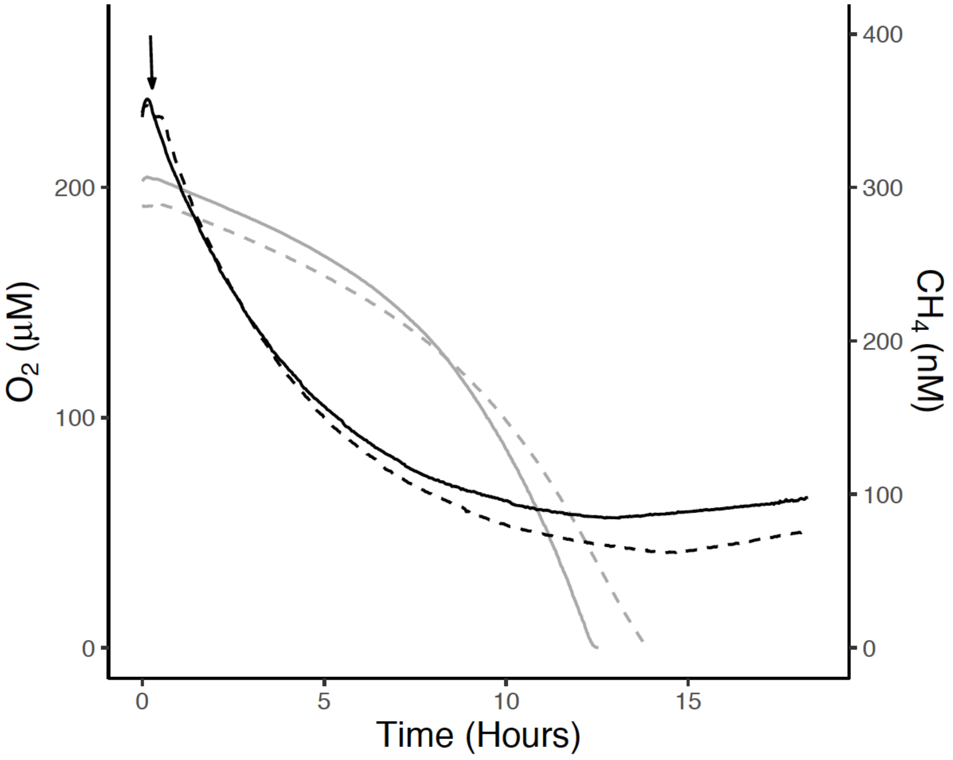

Figure S1. Geochemical data from the November 2021 incubations. Oxygen (grey) and methane (black) concentrations over the course of the two incubations. Data are collected once per minute and represented in line format. Arrow indicates the addition of algal powder. Oxygen consumption is exponential post-addition instead of linear. Methane consumption remains first-order with respect to its concentration.

Table S1. Summary of geochemical and microbial time zero data. Geochemical data from experiments conducted between 10/26/20 through 10/29/21 were first published in Hudspeth *et al.* (2024a).

Date	iBag Replicate #	Geochemistry				Microbial Relative Abundance				
		Rate Constant (hr^{-1})	Modeled Initial Methane (nM)	Modeled Initial Oxygen (µM)	Temperature (°C)	*Methylococcaceae*	*Methylomonadaceae*	*Methylocystaceae*	*Methylophilaceae*	*Methylacidiphilaceae*
2020-10-26	1	0.327	334.07	69.53	20.01	0.0082	0.0043	0.0037	0.0120	0.0243
2020-10-26	2	0.462	334.60	64.40	20.024	0.0089	0.005	0.0030	0.0110	0.0250
2020-10-26	3	0.448	369.88	69.80	20.099	0.0102	0.0049	0.0058	0.0082	0.0202
2021-06-25	1	0.075	331.44	267.80	27.287	0.0011	0.0021	0.0012	0.0049	0.0373
2021-06-25	2	0.087	301.60	275.02	27.308	0.0011	0.0014	0.0009	0.0055	0.0378
2021-06-25	3	0.051	318.03	279.03	27.253	0.0012	0.0008	0.0012	0.0054	0.0346
2021-06-29	1	0.042	378.98	258.31	28.339	0.0004	0.0002	0.0002	0.0063	0.0605
2021-06-29	2	0.029	362.09	274.93	28.544	0.0004	0.0002	0.0004	0.0054	0.0590
2021-06-29	3	0.038	434.36	256.78	28.575	0.0002	0.0001	0.0007	0.0053	0.0505
2021-07-10	1	0.193	195.16	128.08	27.794	0.0014	0.0006	0.0010	0.0034	0.0417

2021-07-10	2	0.238	254.92	133.95	27.93	0.0012	0.0008	0.0007	0.0026	0.0342
2021-10-29	1	0.236	327.93	145.69	20.268	0.0016	0.0003	0.0023	0.0068	0.0256
2021-10-29	2	0.220	333.83	164.36	20.153	0.0020	0.0003	0.0084	0.0034	0.0133
2021-10-29	3	0.231	347.38	155.51	20.127	0.0012	0.0006	0.0029	0.0051	0.0160
2021-11-03	2	0.246	379.15	231.80	18.287	0.0010	0.0001	0.0117	0.0040	0.0159
2021-11-03	3	0.242	408.75	217.12	18.276	0.0007	0.0002	0.0096	0.0035	0.0104

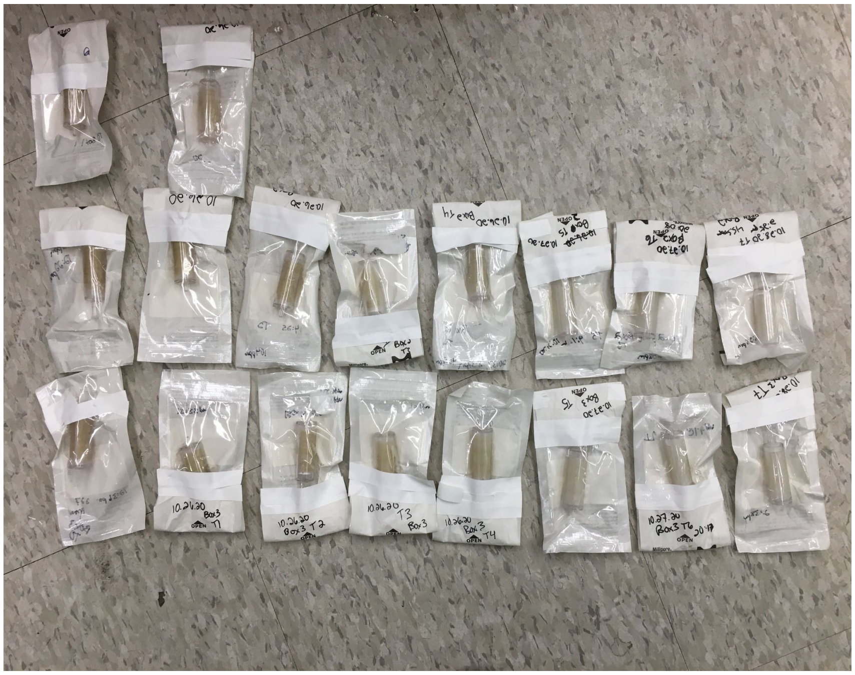

Figure S2. October 2020 Sterivex filters from all three incubations. Top row: incubation 1**, Middle row:** incubation 2, and **Bottom row:** incubation 3 in order of sampling time **(left to right).** The color of the Sterivex filters lightens noticeably as the incubations progress, with the first samples being colored a dark brown and the final ones being off-white. As the incubations are opaque and do not permit light transfer, this loss of pigment could be due to the death and consumption of the photosynthetic organisms within the incubations. Final samples from incubations 2 & 3 at > 300 hours are not otherwise assessed as they were taken long after the incubations turned anoxic.

Table S2. Summary of correlations between the rarefied and non-rarefied 16S rRNA gene amplicon proportion time zero data.

Group	Family	R^2	t	P value
Obligate Methanotroph	*Methylococcaceae*	0.98	24.85	5.57E-13
Obligate Methanotroph	*Methylomonadaceae*	0.98	25.41	4.10E-13
Facultative Methanotroph	*Methylocystaceae*	0.98	29.23	5.97E-14
Nonmethanotrophic Methylotroph	*Methylophilaceae*	0.97	21.26	4.70E-12
Nonmethanotrophic Methylotroph	*Methylacidiphilaceae*	0.995	53.72	< 2.2e-16

Table S3. Summary of correlations between the rarefied and non-rarefied 16S rRNA gene amplicon proportion data for each incubation set.

Incubation Set	Group	Family	R^2	t	P value
October 2020	Obligate Methanotroph	*Methylococcaceae*	0.97	23.44	5.94E-13
	Obligate Methanotroph	*Methylomonadaceae*	0.99	33.94	1.34E-15
	Facultative Methanotroph	*Methylocystaceae*	0.99	32.39	2.68E-15
	Nonmethanotrophic Methylotroph	*Methylophilaceae*	0.99	32.40	2.67E-15
	Nonmethanotrophic Methylotroph	*Methylacidiphilaceae*	0.98	25.40	9.63E-14
October 2021	Obligate Methanotroph	*Methylococcaceae*	0.90	19.18	2.08E-08
	Obligate Methanotroph	*Methylomonadaceae*	0.72	15.87	3.43E-05
	Facultative Methanotroph	*Methylocystaceae*	0.99	30.53	6.99E-15
	Nonmethanotrophic Methylotroph	*Methylophilaceae*	0.98	41.67	5.14E-13
	Nonmethanotrophic Methylotroph	*Methylacidiphilaceae*	0.99	61.52	4.11E-15

November 2021	Obligate Methanotroph	*Methylococcaceae*	0.94	11.27	4.61E-16
	Obligate Methanotroph	*Methylomonadaceae*	0.91	5.97	3.17E-14
	Facultative Methanotroph	*Methylocystaceae*	0.97	34.14	< 2.2e-16
	Nonmethanotrophic Methylotroph	*Methylophilaceae*	0.99	25.00	< 2.2e-16
	Nonmethanotrophic Methylotroph	*Methylacidiphilaceae*	0.99	35.48	< 2.2e-16
June 2021(First)	Obligate Methanotroph	*Methylococcaceae*	0.84	11.27	2.74E-11
	Obligate Methanotroph	*Methylomonadaceae*	0.95	22.14	< 2.2e-16
	Facultative Methanotroph	*Methylocystaceae*	0.94	19.67	< 2.2e-16
	Nonmethanotrophic Methylotroph	*Methylophilaceae*	0.97	27.93	< 2.2e-16
	Nonmethanotrophic Methylotroph	*Methylacidiphilaceae*	0.995	73.08	< 2.2e-16
June 2021 (Second)	Obligate Methanotroph	*Methylococcaceae*	0.50	4.55	0.000174
	Obligate Methanotroph	*Methylomonadaceae*	0.74	7.71	1.48E-07
	Facultative Methanotroph	*Methylocystaceae*	0.85	10.90	4.21E-10
	Nonmethanotrophic Methylotroph	*Methylophilaceae*	0.91	14.32	2.61E-12
	Nonmethanotrophic Methylotroph	*Methylacidiphilaceae*	0.995	67.84	< 2.2e-16
July 2021	Obligate Methanotroph	*Methylococcaceae*	0.99	30.77	1.57E-13
	Obligate Methanotroph	*Methylomonadaceae*	0.81	7.56	4.12E-06
	Facultative Methanotroph	*Methylocystaceae*	0.86	8.81	7.66E-07
	Nonmethanotrophic Methylotroph	*Methylophilaceae*	0.93	13.23	6.42E-09
	Nonmethanotrophic Methylotroph	*Methylacidiphilaceae*	0.99	50.80	2.44E-16

Table S4. qPCR Primers used.

Target Gene	Specificity	Positive Control Amplified	Samples Amplified	Probe/ primer	Sequence (5' – 3')	Reference
pmoA	*Methylacidiphilum*	Yes	No	V170F	GGATWGATTGGAAAGATMG	Sharp *et al.*, 2012
				V613b	GCAAARCTYCTCATYGTWCC	Sharp *et al.*, 2012
pmoA	*Methylococcus*	Yes	Yes	A189F	GGNGACTGGGACTTCTGG	Kolb *et al.*, 2003
				Mc468R	GCSGTGAACAGGTAGCTGCC	Kolb *et al.*, 2003
16s rRNA	Most Bacteria	Yes	Yes	BAC340F	TCCTACGGGAGGCAGCAGT	Kubo *et al.*, 2012
				Bac515R	CGTATTACCGCGGCTGCTGGCAC	Kubo *et al.*, 2012

Table S5. One-way ANOVA comparing the T0 16S rRNA gene amplicon relative abundances of the aerobic methanotrophs and nonmethanotrophic methylotrophs in Jordan Lake by family.

Type	Sum of Squares	df	Mean Square	F	P value
Between Groups	137.37	4	34.34	35.61	< 2E-16
Residuals	72.34	75	0.96		
Total	209.71	79			

Table S6. Tukey's HSD multiple comparisons performed on the one-way ANOVA from Table S5. Confidence interval is abbreviated as CI. Mean difference, standard error, and CI bounds are reported as log_{10} transformed.

Group 1	Group 2	Mean Difference	Standard Error	P value (adjusted)	Lower Bound of CI	Upper Bound of CI
Methylacidiphilaceae	*Methylophilaceae*	1.66	0.93	**8.31E-5**	0.69	2.63
	Methylocystaceae	2.71	0.93	**< 2E-16**	1.74	3.68
	Methylococcaceae	3.00	0.93	**< 2E-16**	2.03	3.97
	Methylomonadaceae	3.80	0.93	**< 2E-16**	2.83	4.77
Methylophilaceae	*Methylocystaceae*	1.05	0.93	**0.028**	0.08	2.02
	Methylococcaceae	1.34	0.93	**0.002**	0.37	2.31
	Methylomonadaceae	2.14	0.93	**3.00 E-7**	1.17	3.11
Methylocystaceae	*Methylococcaceae*	0.30	0.93	0.91	-0.68	1.27
	Methylomonadaceae	1.09	0.93	**0.019**	0.12	2.06
Methylococcaceae	*Methylomonadaceae*	0.80	0.93	0.16	-0.17	1.77

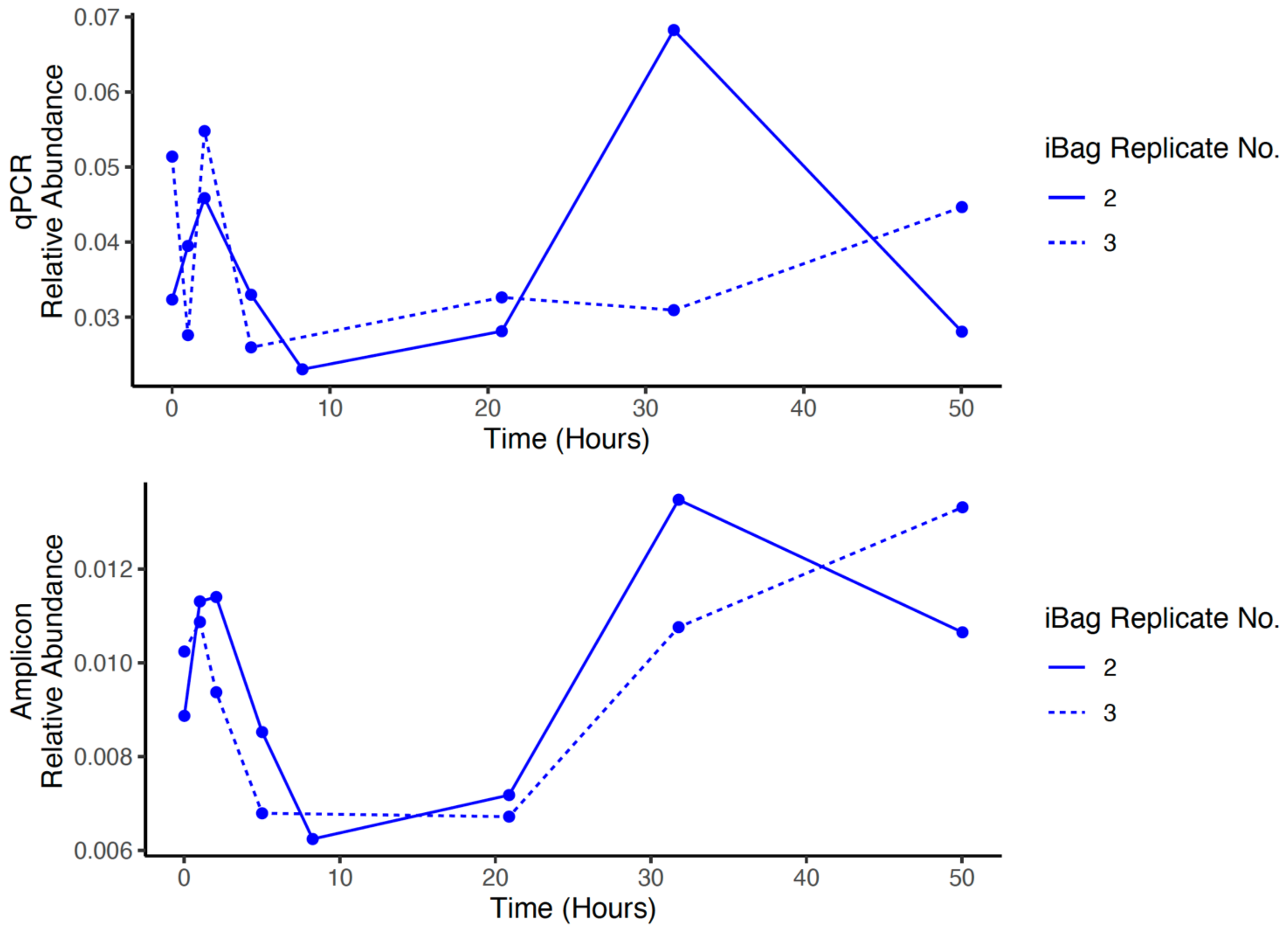

Figure S3. Qualitative comparison of the shifts in relative abundance of *Methylococcaceae* in the October 2020 incubations. Relative abundance calculated from qPCR **(top)** and from 16S rRNA gene amplicon sequencing **(bottom).** Though the correlation between the two datasets only has an R^2 of 0.41 (t = 3.02, df = 13, P = 0.010), the overall trend exhibited is similar across the data sets, indicating that qualitative analysis of the amplicon data set should not be biased.

Table S7. Summary of aerobic methane oxidation (MO_x) rate constant correlations with variables measured at the beginning of each incubation. All Pearson's product-moment correlations have 14 degrees of freedom. Significant correlations are bolded.

Family	Type	R^2	T	P value
Methylococcaceae	**Initial**	**0.73**	**6.20**	**2.32E-05**
	**Average**	**0.47**	**3.50**	**0.004**
Methylomonadaceae	**Initial**	**0.30**	**2.47**	**0.027**
	**Average**	**0.32**	**2.58**	**0.022**
Methylocystaceae	**Initial**	**0.45**	**3.37**	**0.005**
	**Average**	**0.52**	**3.90**	**0.002**
Methylophilaceae	Initial	0.14	1.49	0.16
	Average	0.07	0.99	0.34
Methylacidiphilaceae	**Initial**	**0.38**	**-2.90**	**0.012**
	Average	0.21	-1.94	0.073

Table S8. Summary of geochemical correlations with the qPCR and amplicon relative abundance of *Methylococcaceae.* Results of Pearson product-moment correlation between the qPCR relative abundance of *Methylococcaceae* (*Methylococcaceae pmoA* gene copies / Bacterial 16S rRNA gene copies) and the three geochemical variables (oxygen, methane, and temperature) along with the first-order rate constant of MO_x. Correlations have 10 degrees of freedom. Statistically significant correlations are bolded.

Variable	R^2	t	P value
**Oxygen**	**0.83**	**-7.03**	**3.57E-05**
Methane	0.08	-0.92	0.38
**Temperature**	**0.63**	**-4.12**	**2.07E-03**
**MO_x Rate Constant**	**0.93**	**11.69**	**3.74E-07**

Table S9. Significant correlations between the first-order rate constant of MO_x and the 16S rRNA gene amplicon relative abundance of microbial families in Jordan Lake. Of the 407 total families identified in Jordan Lake and of the 116 families in turn present in all T0 samples, 38 exhibit significant correlations.

Family	R	T	P value
Methylococcaceae	0.8561367	6.20	2.32E-05
Neisseriaceae	0.84602511	5.94	3.62E-05
cvE6	0.84268009	5.86	4.17E-05
Isosphaeraceae	0.81561236	5.27	1.18E-04
Diplorickettsiaceae	0.80952935	5.16	1.45E-04
B1-7BS	0.79043696	4.83	2.68E-04
A4b	0.78940101	4.81	2.77E-04
Prochlorotrichaceae	0.7718399	4.54	4.61E-04
Hydrogenedensaceae	0.75689013	4.33	6.88E-04
Opitutaceae	0.72593268	3.95	1.45E-03
Oligoflexaceae	0.71196395	3.79	1.98E-03
Steroidobacteraceae	0.70634089	3.73	2.22E-03
Ilumatobacteraceae	0.69633502	3.63	2.73E-03
Beijerinckiaceae	0.68877046	3.55	3.17E-03
TRA3-20	0.6791363	3.46	3.81E-03
Vicinamibacteraceae	0.67340154	3.41	4.24E-03
Legionellaceae	0.64001552	3.12	7.58E-03

Holosporaceae	0.62346599	2.98	9.87E-03
Polyangiaceae	0.61777031	2.94	0.0108
Saprospiraceae	0.61643997	2.93	0.0110
Rhodocyclaceae	0.60824822	2.87	0.0124
Gemmataceae	0.58469196	2.70	0.0174
Methylomonadaceae	0.55132638	2.47	0.0268
Microcystaceae	0.49762258	2.15	0.0498
Burkholderiaceae	-0.4992541	-2.16	0.0490
LiUU-11-161	-0.5048546	-2.19	0.0461
Unknown Family	-0.5246969	-2.31	0.0369
Mycoplasmataceae	-0.5368755	-2.38	0.0320
Methylacidiphilaceae	-0.6131463	-2.90	0.0115
Leptolyngbyaceae	-0.6136544	-2.91	0.0115
Acetobacteraceae	-0.651889	-3.22	6.21E-03
MWH-UniP1 aquatic group	-0.7338908	-4.04	1.21E-03
Cyanobiaceae	-0.7366352	-4.08	1.14E-03
Terrimicrobiaceae	-0.7585908	-4.37	6.58E-03
Oxalobacteraceae	-0.7853458	-4.75	3.12E-04
Nostocaceae	-0.8243814	-5.45	8.57E-05
Nodosilineaceae	-0.8388652	-5.77	4.88E-05
Pseudanabaenaceae	-0.8544915	-6.15	2.50E-05

Table S10. Significant correlations between the first-order rate constant of MO_x and the 16S rRNA gene amplicon relative abundance of microbial genera in Jordan Lake. Out of 735 total genera identified in Jordan and of the 140 genera in turn present in all T0 samples, 32 exhibit significant correlations.

Genus	R	t	P value
Methyloparacoccus	0.882435537	7.02	6.07E-06
Prochlorothrix PCC-9006	0.771839901	4.54	4.61E-04
Opitutus	0.748809423	4.23	8.45E-04
CL500-29 marine group	0.701870028	3.69	2.44E-03
Methylocystis	0.668937274	3.67	4.60E-03
Rhodopirellula	0.665939758	3.34	4.86E-03
MND1	0.664578169	3.33	4.98E-03
Silvanigrella	0.663826561	3.32	5.05E-03
Candidatus Aquirestis	0.659680741	3.28	5.43E-03
DEV114	0.632667687	3.06	8.53E-03
oc32	0.602660163	2.83	0.0135
Legionella	0.593598619	2.76	0.0153
Microcystis PCC-7914	0.567177404	2.58	0.0219
alphaI cluster	0.564028181	2.56	0.0229
Rubrivivax	-0.541203689	-2.41	0.0304
Aphanizomenon NIES81	-0.593401982	-2.76	0.0154
Sphaerospermopsis BCCUSP55	-0.638271964	-3.10	7.80E-03

Leptospira	-0.644004888	-3.15	7.09E-03
Sphaerotilus	-0.651289997	-3.21	6.28E-03
Edaphobaculum	-0.665606468	-3.34	4.89E-03
SM1A02	-0.73474053	-4.05	1.19E-03
Cyanobium PCC-6307	-0.736635225	-4.08	1.14E-03
Nodularia PCC-9350	-0.749552649	-4.24	8.29E-04
Terrimicrobium	-0.759321224	-4.37	6.46E-04
Pirellula	-0.774984804	-4.59	4.22E-04
Cuspidothrix LMECYA 163	-0.80373063	-5.05	1.76E-04
Roseomonas	-0.810122411	-5.17	1.42E-04
DSSD61	-0.838431824	-5.76	4.97E-05
Nodosilinea PCC-7104	-0.838865242	-5.77	4.88E-05
Cylindrospermopsis CRJ1	-0.844449994	-5.90	3.88E-05
Pseudanabaena PCC-7429	-0.854491522	-6.15	2.450E-05
Noviherbaspirillum	-0.893816213	-7.46	3.07E-06

Table S11. Significant correlations between the initial MO_x rate and the 16S rRNA gene amplicon relative abundance of microbial families in Jordan Lake. Of the 407 total families identified in Jordan Lake and of the 116 families in turn present in all T0 samples, 37 exhibit significant correlations.

Family	R	t	P value
Isosphaeraceae	0.86450145	6.44	1.56E-05
cvE6	0.84051108	5.80	4.57E-05
Methylococcaceae	0.80896622	5.15	1.48E-04
Prochlorotrichaceae	0.79749045	4.95	2.15E-04
Hydrogenedensaceae	0.79040008	4.84	2.68E-04
Oligoflexaceae	0.78365502	4.72	3.28E-04
A4b	0.77829492	4.64	3.84E-04
Neisseriaceae	0.75749812	4.34	6.77E-04
Opitutaceae	0.75181564	4.27	7.83E-04
Diplorickettsiaceae	0.74321178	4.16	9.69E-04
Beijerinckiaceae	0.73067652	4.00	1.30E-03
B1-7BS	0.70597643	3.73	2.24E-03
Steroidobacteraceae	0.69205516	3.59	2.97E-03
Legionellaceae	0.67135001	3.39	4.41E-03
Holosporaceae	0.66117221	3.30	5.29E-03
Gemmataceae	0.65535253	3.25	5.85E-03
Vicinamibacteraceae	0.63446984	3.07	8.29E-03

Ilumatobacteraceae	0.5997038	2.80	0.0141
TRA3-20	0.5934531	2.76	0.0154
Saprospiraceae	0.56202064	2.54	0.0235
Microcystaceae	0.53963516	2.40	0.0310
Rhodocyclaceae	0.53669037	2.38	0.0321
Polyangiaceae	0.5097301	2.22	0.0437
LiUU-11-161	-0.5248616	-2.30	0.0368
Mycoplasmataceae	-0.5544397	-2.49	0.0258
Burkholderiaceae	-0.6034626	-2.83	0.0133
Hyphomonadaceae	-0.6189949	-2.95	0.0106
Leptolyngbyaceae	-0.6540407	-3.24	5.99E-03
Methylacidiphilaceae	-0.6801416	-3.47	3.74E-03
Cyanobiaceae	-0.6945727	-3.61	2.83E-03
Acetobacteraceae	-0.708657	-3.76	2.12E-03
Terrimicrobiaceae	-0.714207	-3.82	1.88E-03
MWH-UniP1 aquatic group	-0.8035337	-5.05	1.77E-04
Nodosilineaceae	-0.8261986	-5.49	8.00E-05
Oxalobacteraceae	-0.8377201	-5.74	5.12E-05
Nostocaceae	-0.8750697	-6.76	9.10E-06
Pseudanabaenaceae	-0.8901377	-7.31	3.86E-06

Table S12. Significant correlations between the initial MO_x rate and the 16S rRNA gene amplicon relative abundance of microbial genera in Jordan Lake. Out of 735 total genera identified in Jordan and of the 140 genera in turn present in all T0 samples, 34 exhibit significant correlations.

Genus	R	t	P value
Methyloparacoccus	0.84024577	5.80	4.62E-05
Prochlorothrix PCC-9006	0.79749045	4.95	2.15E-04
Opitutus	0.77972882	4.66	3.68E-04
Methylocystis	0.73638842	4.07	1.14E-03
Silvanigrella	0.72764852	3.97	1.40E-03
Legionella	0.63223376	3.05	8.59E-03
Candidatus Aquirestis	0.61988673	2.96	0.0104
CL500-29 marine group	0.59914241	2.80	0.0142
Microcystis PCC-7914	0.59091139	2.74	0.0159
DEV114	0.57556802	2.63	0.0197
alphaI cluster	0.56631521	2.57	0.0221
Rhodopirellula	0.54752221	2.45	0.0281
MND1	0.52196534	2.29	0.0381
Limnobacter	-0.5000882	-2.16	0.0485
Acidibacter	-0.5346594	-2.37	0.0329
Hirschia	-0.5989279	-2.80	0.0142
Rubrivivax	-0.6098454	-2.88	0.0121

Edaphobaculum	-0.6341623	-3.07	8.33E-03
Aphanizomenon NIES81	-0.64783	-3.18	6.66E-03
Sphaerospermopsis BCCUSP55	-0.6862019	-3.53	3.33E-03
Cyanobium PCC-6307	-0.6945727	-3.61	2.83E-03
Terrimicrobium	-0.716921	-3.85	1.78E-03
Leptospira	-0.7444697	-4.17	9.40E-04
Sphaerotilus	-0.7642595	-4.43	5.66E-04
Pirellula	-0.7654479	-4.45	5.49E-04
SM1A02	-0.7895189	-4.81	2.76E-04
Nodularia PCC-9350	-0.8206481	-5.37	9.82E-05
Nodosilinea PCC-7104	-0.8261986	-5.49	8.00E-05
Roseomonas	-0.8542443	-6.15	2.53E-05
Cuspidothrix LMECYA 163	-0.8641582	-6.43	1.59E-05
Cylindrospermopsis CRJ1	-0.8856417	-7.14	5.04E-06
Pseudanabaena PCC-7429	-0.8901377	-7.31	3.86E-06
DSSD61	-0.8932206	-7.43	3.19E-06
Noviherbaspirillum	-0.9237936	-9.03	3.27E-07

Table S13. Summary of correlations between the geochemical parameters and 16S rRNA gene amplicon relative abundance of key families. Results of Pearson product-moment correlation between the 16S rRNA gene amplicon relative abundance of the key families and the three geochemical variables. Correlations have 14 degrees of freedom. Statistically significant correlations are bolded.

Family	Variable	R^2	t	P value
Methylococcaceae, obligate methanotroph	**Oxygen**	**0.69**	**-5.52**	**7.57E-05**
	Methane	0.08	-1.08	0.30
	**Temperature**	**0.31**	**-2.51**	**0.025**
Methylomonadaceae, obligate methanotroph	**Oxygen**	**0.35**	**-2.72**	**0.017**
	Methane	0.12	-1.37	0.19
	Temperature	0.01	-0.45	0.66
Methylocystaceae, facultative methanotroph	Oxygen	0.15	-1.78	0.097
	Methane	0.04	0.76	0.46
	**Temperature**	**0.84**	**-8.43**	**7.39E-07**
Methylophilaceae, nonmethanotrophic methylotroph	Oxygen	0.14	-1.49	0.16
	Methane	0.07	1.02	0.32
	Temperature	0.06	-0.93	0.37
Methylacidiphilaceae, nonmethanotrophic methylotroph (inferred)	Oxygen	0.16	1.59	0.13
	Methane	0.28	-0.74	0.47
	**Temperature**	**0.81**	**7.81**	**1.82E-06**

Table S14. Summary of model statistics of the best linear models for each family.

Family	Adjusted R^2	F	Df	P
Methylococcaceae	0.8915	42.08	3, 12	1.21E-06
Methylomonadaceae	0.7262	10.95	4, 11	7.89E-04
Methylocystaceae	0.8355	71.1	1, 14	7.39E-07
Methylophilaceae	0.8072	21.93	2, 13	3.68E-05
Methylacidiphilaceae	0.853	30.01	3, 12	7.36E-06

Table S15. Summary of all multiple regression results of the best linear models for each family.

Family	Predictor	Standardized Slope ± SE	F	df	P value
Methylococcaceae	Oxygen	1.936e-02 ± 4.355e-03	84.20	1, 12	8.99E-07
	Methane	1.214e-02 ± 2.337e-03	0.14	1, 12	0.72
	Oxygen:Methane	-7.089e-05 ± 1.265e-05	31.40	1, 12	1.15E-04
Methylomonadaceae	Oxygen	3.492e-02 ± 8.740e-03	12.88	1, 11	4.25E-03
	Methane	2.265e-02 ± 5.315e-03	0.14	1, 11	0.72
	Oxygen:Methane	-1.225e-04 ± 2.640e-05	21.53	1, 11	7.26E-04
	Temperature	8.202e-02 ± 2.827e-02	8.41	1, 11	0.014
Methylocystaceae	Temperature	-0.10905 ± 0.01293	71.10	1.14	7.39E-07
Methylophilaceae	Oxygen	-1.485e-02 ± 1.923e-03	6.27	1, 12	0.028
	Temperature	-1.222e-01 ± 1.714e-02	0.18	1, 12	0.68
	Oxygen:Temperature	6.005e-04 ± 8.098e-05	54.98	1, 12	8.11E-06
Methylacidiphilaceae	Oxygen	-5.376e-03 ± 2.121e-03	1.42	1, 12	0.26
	Temperature	1.010e-02 ± 1.891e-02	68.76	1, 12	2.60E-06
	Oxygen:Temperature	2.121e-04 ± 8.932e-05	5.64	1, 12	0.035

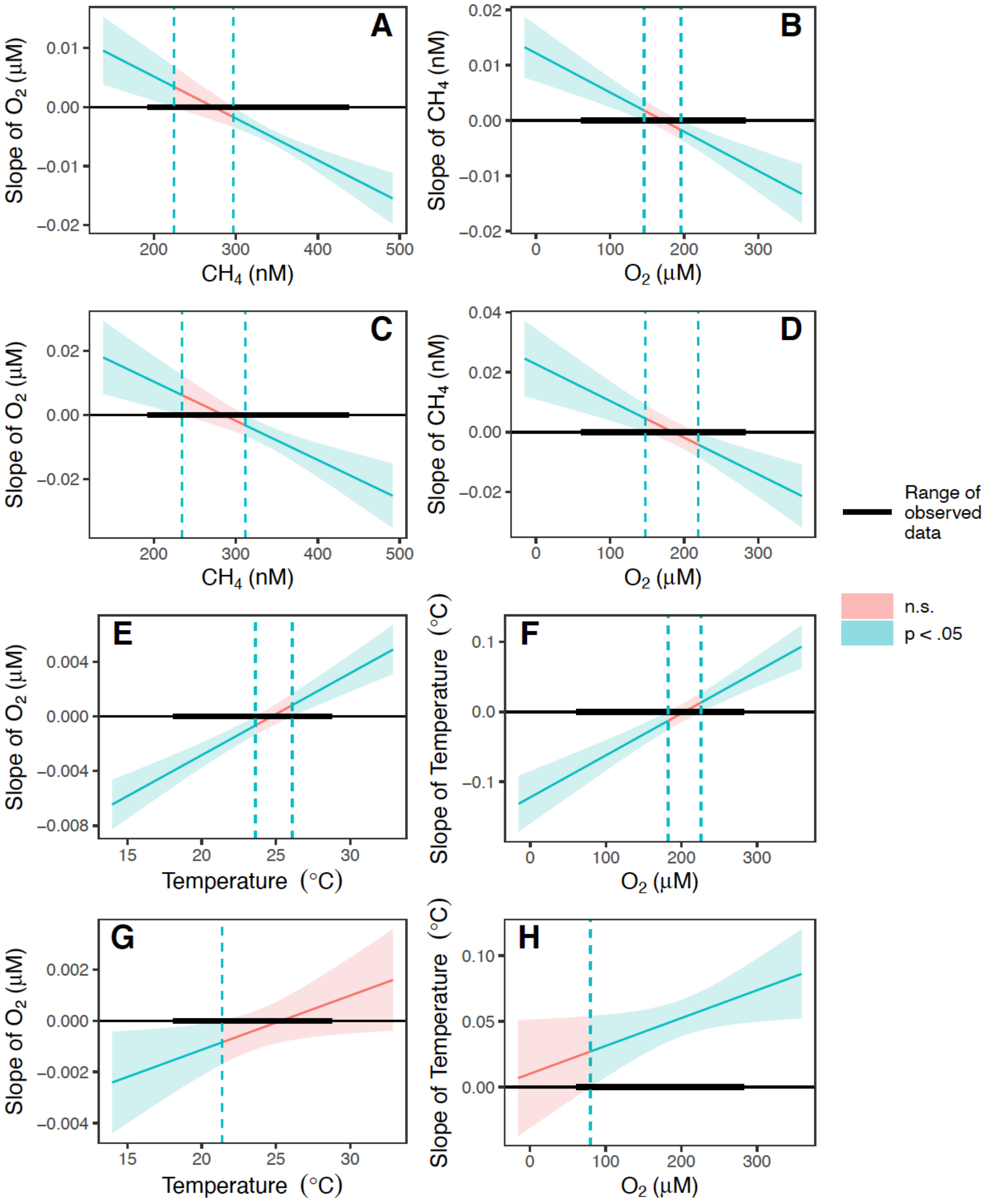

Figure S4. **Johnson-Neyman interval plots for the multiple regressions with continuous-by-continuous interaction terms.** The calculated slopes and 95% Cis in blue are significant ($P < 0.05$) and those in red are not. Oxygen and methane interactions in the *Methylococcaceae* model **(A, B)**. Oxygen and methane interactions in the *Methylomonadaceae* model **(C, D)**. Oxygen and temperature interaction in the *Methylophilaceae* model **(E, F)**. Oxygen and temperature interaction in the *Methylacidiphilaceae* model **(G, H)**.

Table S16. Summary statistics of each model in the pool of possible models for each family. Best models are bolded. Residual degrees of freedom apply to each specific term added to the model. The corrected Akaike information criterion (AICc) and the Bayesian information criterion (BIC) are presented. The best models had the lowest AICc and/or BIC, often having the highest adjusted R^2 value as well.

Family	Model	Residual df	Adjusted R^2	AICc	BIC
Methylomonadaceae	Intercept	1,15	--	31.77	32.39
	Oxygen	1,14	0.2995	28.04	28.36
	Methane	1,14	0.0559	32.82	33.16
	Temperature	1,14	-0.06	34.61	34.93
	Oxygen + Methane	1,13	0.28	30.89	30.35
	Oxygen + Temperature	1,13	0.31	30.21	29.66
	Methane + Temperature	1,13	0.04	35.48	34.93
	Oxygen + Methane + (O2:CH4)	1,12	0.56	26.24	24.11
	Oxygen + Temperature + (O2:Temperature)	1,12	0.50	28.19	26.06
	Oxygen + Methane + Temperature	1,12	0.26	34.50	32.36
	**Oxygen + Methane + Temperature + (O2:CH4)**	**1,11**	**0.73**	**22.49**	**17.79**
	Oxygen + Methane + Temperature + (O2:Temperature)	1,11	0.63	27.38	22.68
Methylocystaceae	Intercept	1,15	--	27.96	28.58
	Oxygen	1,14	0.13	27.77	28.09
	Methane	1,14	-0.03	30.39	30.70
	**Temperature**	**1,14**	**0.82**	**2.16**	**2.48**
	Oxygen + Methane	1,13	0.20	28.90	28.35
	Oxygen + Temperature	1,13	0.82	5.16	4.61
	Methane + Temperature	1,13	0.82	5.34	4.80
	Oxygen + Methane + Temperature	1,12	0.8262	7.47	5.33

Methylococcaceae	Intercept	1,15	--	25.45	26.07
	Oxygen	1,14	0.66	10.04	10.38
	Methane	1,14	0.01	27.25	27.57
	Temperature	1,14	0.26	22.59	22.92
	Oxygen + Methane	1,13	0.64	13.63	13.08
	Oxygen + Temperature	1,13	0.66	12.84	12.29
	Methane + Temperature	1,13	0.45	20.42	19.87
	**Oxygen + Methane + (O2:CH4)**	**1,12**	**0.89**	**-2.58**	**-4.71**
	Oxygen + Temperature + (O2:Temperature)	1,12	0.73	12.25	10.12
	Oxygen + Methane + Temperature	1,12	0.65	16.35	14.21
	Oxygen + Methane + Temperature + (O2:CH4)	1,11	0.89	2.75	-1.94
	Oxygen + Methane + Temperature + (O2:Temperature)	1,11	0.86	5.66	0.96
Methylacidiphilaceae	Intercept	1,15	--	2.33	2.96
	Oxygen	1,14	0.09	2.75	3.07
	Methane	1,14	-0.03	4.79	5.11
	Temperature	1,14	0.80	-21.43	-21.11
	Oxygen + Methane	1,13	0.15	4.23	3.69
	Oxygen + Temperature	1,13	0.80	-19.04	-19.58
	Methane + Temperature	1,13	0.79	-18.22	-18.76
	**Oxygen + Temperature + (O2:Temperature)**	**1,12**	**0.85**	**-20.83**	**-22.97**
	Oxygen + Methane + Temperature	1,12	0.82	-17.46	-19.60
	Oxygen + Methane + Temperature + (O2:Temperature)	1,11	0.84	-15.75	-20.45

Methylophilaceae	Intercept	1,15	--	-5.14	-4.52
	Oxygen	1,14	0.07505	-4.41	-4.10
	Methane	1,14	0.002836	-3.21	-2.89
	Temperature	1,14	-0.008722	-3.03	-2.71
	Oxygen + Methane	1,13	0.1809	-3.91	-4.45
	Oxygen + Temperature	1,13	0.006624	-0.82	-1.37
	Methane + Temperature	1,13	-0.04	-0.10	-0.64
	Methane + Temperature + (CH4:Temperature)	1,12	0.27	-2.70	-4.84
	**Oxygen + Temperature + (O2:Temperature)**	**1,12**	**0.81**	**-23.97**	**-26.10**
	Oxygen + Methane + Temperature	1,12	0.17	-0.56	-2.70
	Oxygen + Methane + Temperature + (CH4:Temperature)	1,11	0.33	0.01	-4.68
	Oxygen + Methane + Temperature + (O2:Temperature)	1,11	0.79	-18.74	-23.44

Table S17. Correlations between geochemical predictors. None exhibited collinearity as defined in the methods. All had 14 degrees of freedom.

Predictors	R^2	T	P value
Oxygen & Temperature	0.30	2.43	0.029
Oxygen & Methane	0.09	1.17	0.26
Methane & Temperature	0.08	-1.13	0.28

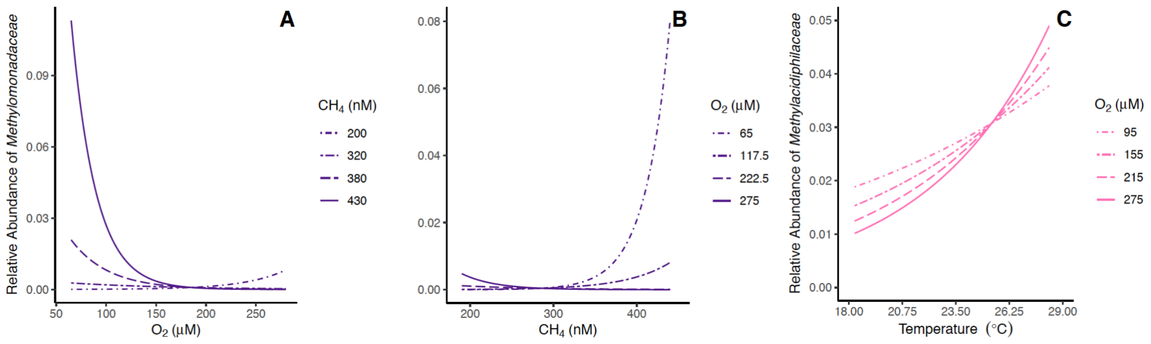

Figure S5. Predicted effects of continuous-by-continuous two-way interactions on *Methylomonadaceae* and *Methylacidiphilaceae*. The predicted effect of oxygen modulated by methane and methane modulated by oxygen, respectively, on *Methylomonadaceae* 16S rRNA gene amplicon relative abundance **(A,B)**. Temperature is set to 19.656°C, the average temperature recorded at the start of the fall incubations. The predicted effect of temperature modulated by oxygen on *Methylacidiphilaceae* 16S rRNA gene amplicon relative abundance **(C)**. The predicted effect of oxygen modulated by temperature on *Methylacidiphilaceae* 16S rRNA gene amplicon relative abundance is not shown, as oxygen significantly affects the 16S rRNA gene amplicon abundance for only ~3 degrees of the 10.3°C range of temperatures observed in this study and the effect does not meaningfully vary within that range.

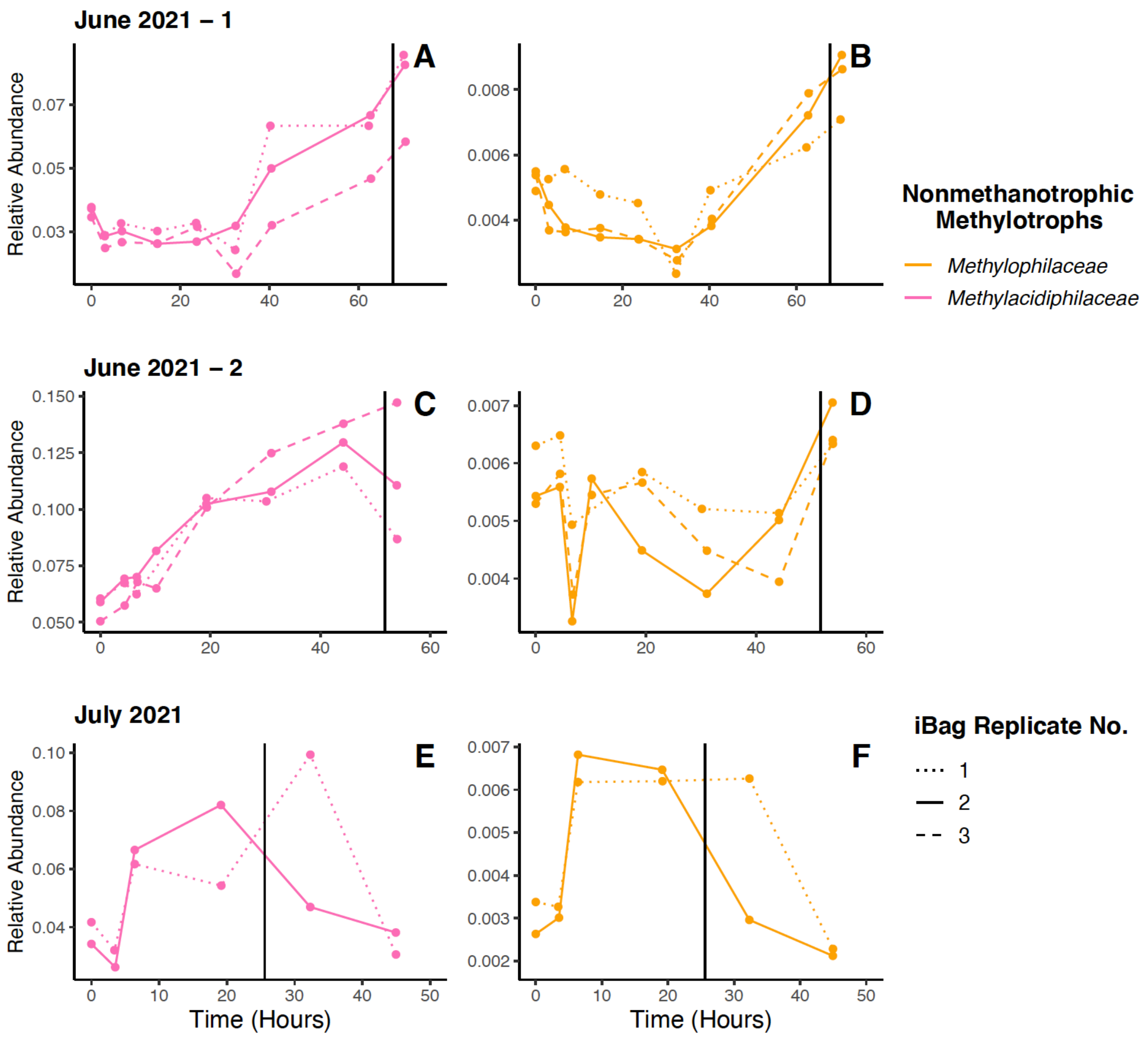

Figure S6. 16S rRNA gene amplicon relative abundance data for nonmethanotrophic methylotrophs from the summer incubations. Three sets of *in situ* incubations were performed during the Summer months: one in June 2021 **(A,B)**, a second in June 2021 **(C,D)**, and one in July 2021 **(E,F)**. Incubations ended shortly after becoming anoxic. Immediately after starting the incubations, ammonium was added to the first and third iBags deployed, with the second serving as a control. The iBags that received ammonium in the first June 2021 experiment received ~21 µM and those that did in the other two experiments received ~42 µM. The 16S rRNA gene amplicon relative abundance of *Methylacidiphilaceae* **(A,C,E)** and *Methylophilaceae* **(B,D,F)** are shown. Vertical lines indicate when oxygen concentrations fell below 20 µM.

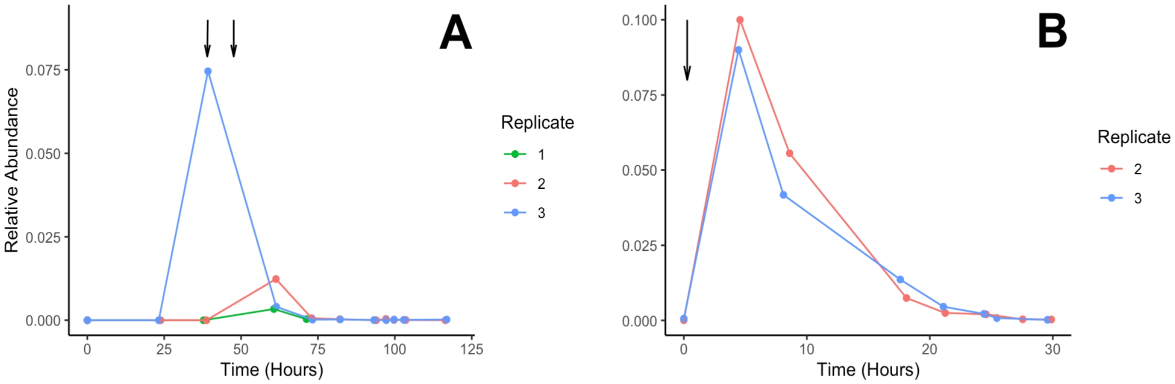

Figure S7. 16S rRNA gene amplicon relative abundance of the dried algae (*Arthrospira PCC-7345*). 16S rRNA gene amplicon sequences over time in the October 2021 (**A**) and the November 2021 incubation sets (**B**). The 16S rRNA gene amplicon relative abundance spikes immediately after addition and then is consumed at an increasing rate over time, nearly vanishing over the course of each incubation. **(A)** First arrow indicates when two capsules were added to the incubations at ~39.16 hours. This occurred prior to sampling for replicates 1 and 2, but before sampling for replicate 3. The second arrow indicates when ten capsules were added at ~47.61 hours for all three replicates. **(B)** The arrow indicates when twelve capsules were added at ~0.2750 hours.

References

Hudspeth, Z.W., Morningstar, J.L., Mendlovitz, H.P., Baily, J.A., Lloyd, K.G., and Martens, C.S. (2024). In situ aerobic methane oxidation rates in a stratified lake. *Limnology and Oceanography*. doi: 10.1002/lno.12583.

Kolb, S., Knief, C., Stubner, S., and Conrad, R. (2003). Quantitative detection of methanotrophs in soil by novel pmoA-targeted real-time PCR assays. *Appl Environ Microbiol* 69(5)**,** 2423-2429. doi: 10.1128/AEM.69.5.2423-2429.2003.

Kubo, K., Lloyd, K.G., J, F.B., Amann, R., Teske, A., and Knittel, K. (2012). Archaea of the Miscellaneous Crenarchaeotal Group are abundant, diverse and widespread in marine sediments. *ISME J* 6(10)**,** 1949-1965. doi: 10.1038/ismej.2012.37.

Sharp, C.E., Stott, M.B., and Dunfield, P.F. (2012). Detection of autotrophic verrucomicrobial methanotrophs in a geothermal environment using stable isotope probing. *Front Microbiol* 3**,** 303. doi: 10.3389/fmicb.2012.00303.
